# Encapsulation of Polyphenolic Compounds Based on Hemicelluloses to Enhance Treatment of Inflammatory Bowel Diseases and Colorectal Cancer

**DOI:** 10.3390/molecules28104189

**Published:** 2023-05-19

**Authors:** Miłosz Caban, Urszula Lewandowska

**Affiliations:** Department of Biochemistry, Faculty of Medicine, Medical University of Lodz, 92-215 Lodz, Poland; milosz.caban@stud.umed.lodz.pl

**Keywords:** bioavailability, bioaccessibility, emulsion, gels, glucan, mannan, microcapsules, particles, polyphenol, xylan

## Abstract

Inflammatory bowel diseases (IBD) and colorectal cancer (CRC) are difficult to cure, and available treatment is associated with troubling side effects. In addition, current therapies have limited efficacy and are characterized by high costs, and a large segment of the IBD and CRC patients are refractive to the treatment. Moreover, presently used anti-IBD therapies in the clinics are primarily aimed on the symptomatic control. That is why new agents with therapeutic potential against IBD and CRC are required. Currently, polyphenols have received great attention in the pharmaceutical industry and in medicine due to their health-promoting properties. They may exert anti-inflammatory, anti-oxidative, and anti-cancer activity, via inhibiting production of pro-inflammatory cytokines and enzymes or factors associated with carcinogenesis (e.g., matrix metalloproteinases, vascular endothelial growth factor), suggesting they may have therapeutic potential against IBD and CRC. However, their use is limited under both processing conditions or gastrointestinal interactions, reducing their stability and hence their bioaccessibility and bioavailability. Therefore, there is a need for more effective carriers that could be used for encapsulation of polyphenolic compounds. In recent years, natural polysaccharides have been proposed for creating carriers used in the synthesis of polyphenol encapsulates. Among these, hemicelluloses are particularly noteworthy, being characterized by good biocompatibility, biodegradation, low immunogenicity, and pro-health activity. They may also demonstrate synergy with the polyphenol payload. This review discusses the utility and potential of hemicellulose-based encapsulations of polyphenols as support for treatment of IBD and CRC.

## 1. Introduction

Inflammatory bowel diseases (IBD) and colorectal cancer (CRC), including IBD-associated CRC, are serious conditions that present global challenges for medicine. The two main subtypes of IBD, ulcerative colitis (UC) and Crohn’s disease (CD), are chronic, progressive, immune-mediated diseases associated with a number of complications and significantly reduced quality of life. Data indicate that the incidence of IBD is increasing, and an exponential increase is anticipated worldwide [1,2]. It is estimated that there will be a 1.8–2.6-fold increase in the prevalent IBD population by 2030 depending on the region compared to 2010 [3]. In addition, IBD often affects young people; approximately 25% of patients with IBD are younger than 20 years, resulting in growth impairment and pubertal delays [4]. Moreover, the chronic colonic inflammation occurring in IBD increases the risk of CRC development depending on the disease duration or individual risk factors. The cumulative risk of CRC in UC patients is estimated to be 2%, 8%, and 18% after 10, 20, and 30 years of disease, respectively [5,6,7]. Nevertheless, IBD is not present in all patients with CRC. Recent data indicate that CRC is the second most common cancer diagnosed in women and third most in men, and the second leading cause of cancer-related deaths globally, representing about 10% of all cases. Approximately 1.8 million new CRC cases are recorded worldwide each year, and this value is predicted to increase to 2.5 million by the year 2035 [8,9]. In contrast, other data present that the number of new cases with CRC in the world will be approximately 4 million by the year 2040, representing an over two-fold rise of prevalence compared to the morbidity from the year 2020 [10]. Unfortunately, the treatment of IBD and CRC is not satisfactory. The medications used in IBD are associated with high costs, may cause a lot of side effects, and their full desirable effect is not often achieved [11,12,13]. In turn, 20% of CRC patients have metastatic disease at the time of diagnosis, precluding the possibility of tumor resection or cure; in such cases, only limited therapeutic options are available, mainly chemo- and radiotherapy, which are associated with many side effects. Furthermore, approximately 40% of CRC cases with stage II-III demonstrate recurrence in the five years after surgical treatment [14]. As such, new therapeutic options are being sought for inducing and maintaining the IBD remission and preventing the development of CRC.

Polyphenols, important compounds in fruits and vegetables, seem to be promising agents, having been found to demonstrate a range of pro-health properties [15,16]. There are four main classes of polyphenolic compounds: flavonoids, phenolic acids, lignans, and stilbenes. Flavonoids, including flavonols, flavanones, isoflavones, anthocyanins, and flavan-3-ols, are the most examined polyphenolic compounds with a lot of pro-health activities, including anti-inflammatory and anti-cancer [15]. The most recent data show that polyphenols are able to ameliorate colon damage, restore disturbed composition of gut microbiota and even inhibit the colonic production of pro-inflammatory mediators occurring in IBD. Additionally, they are able to stimulate the expression of anti-inflammatory cytokines and antioxidant enzymes or limit apoptosis of the epithelial cells of bowels [17]. Polyphenolic compounds also appear to be effective at chemoprevention or inhibiting the development and progression of CRC. They may prevent the development of tumour angiogenesis modulating expression and activity of some of factors and cellular pathways associated with this process, such as vascular endothelial growth factor (VEGF), hypoxia-inducible factor 1 alpha (HIF-1α), protein kinase B (Akt) pathway, and extracellular signal-regulated kinase (ERK) pathway. In addition, polyphenols may downregulate the expression and activity of matrix metalloproteinases (MMPs), mainly MMP-2 and MMP-9, responsible for the cell invasion and the formation of metastases in the CRC disease [18,19,20]. Despite many pre-clinical studies concerning anti-inflammatory and anti-cancer properties of polyphenols in IBD and CRC, few clinical studies have assessed their effectiveness, and their therapeutic effects have not been fully confirmed in clinical trials [17,21,22,23,24]. This may be due to the limitations associated with the low bioavailability and bioaccessibility of polyphenols.

However, it has been found that encapsulation of polyphenols, using micro- and nanocapsules, micro- and nano-emulsions, micro- and nanoparticles, or even more complex delivery systems, may overcome these limitations and improve the effectiveness of these compounds [25]. It is important to emphasize that encapsulates based on compounds having pro-health properties could enhance the activity of polyphenols or exert a stronger and wider spectrum of action. These requirements can be fulfilled by hemicelluloses, which are characterized by low toxicity and biological pro-health activities, including anti-inflammatory and anti-cancer effects [26,27,28,29]. Hence, this review discusses the impact of hemicellulose encapsulation of polyphenolic compounds against IBD and CRC to gain a clearer understanding of its therapeutic potential against these significant civilization diseases.

## 2. Limitations of the Use of Polyphenols in IBD and CRC

### 2.1. Obtaining and Stability of Polyphenols

A few factors may hinder the extraction of natural polyphenols. Firstly, polyphenols may occur in plants as compounds complexed with carbohydrates, proteins, or polymerized derivatives, with increased resistance to effective isolation. Additionally, polyphenols are susceptible to oxidation, which determines the choice of appropriate extraction technique, the extraction time and temperature, the type of solvent, and the ratio of solvent to solid. Moreover, the polyphenol content of the plants may demonstrate seasonal variability. Additionally, it is important to emphasize that different types of polyphenols have varying stability, and consequently, individual compounds require various storage methods and durations and even protection against light [30].

### 2.2. Impact of Digestive Processes and Intestinal Barrier

It must be emphasized that IBD patients are characterized by different gastrointestinal environments to healthy subjects. Firstly, patients with IBD have a lower colonic pH, i.e., 2.3–5.5, compared to 6.0–6.5 in the healthy colon, inducing changes in absorption, local aggregation, or chemical modifications of polyphenols; this results from altered lactic acid and bicarbonate production by microbiota and mucosal absorption of short-chain fatty acids (SCFA) [31]. Secondly, gut dysmotility is observed in IBD [32], with the transport time through the gastrointestinal tract to be decreased compared to subjects without colonic inflammation (24 h vs. 6–70 h); this may prevent the polyphenols from completing their activity and hasten their clearance from the inflamed colon [33]. Thirdly, the inflammatory colonic response occurring in IBD damages the epithelium, thus increasing intestinal barrier permeability. Such damage results from intense apoptosis of epithelial cells, diminished expression of tight junction proteins or degradation and production dysregulation of mucus, leading to the presence of numerous positively-charged proteins that may electrostatically interact with other molecules, including polyphenols [34,35,36].

Interestingly, individual groups of polyphenolic compounds differ from each other with regard to the localization of absorption and structural modifications. For example, anthocyanins are mainly absorbed in the acidic pH of the stomach, where they have a flavylium cations structure. In contrast, carbinol forms dominate in the intestinal environment, significantly limiting absorption [37]. Hence, it is important to carefully choose the correct polyphenol for treatment in the inflamed part of the digestive tract. Therefore, anthocyanins or other polyphenols absorbed in the acidic environment appear suitable for therapy of CD, in which both the colon and the stomach may be affected [38].

### 2.3. Disturbances of Gut Microbiota

Although the gut microbiota is necessary to maintain intestinal homeostasis and function, it plays a key role in the metabolism of polyphenols by enabling their deconjugation, hydrolysis, and even degradation [39]. It is worth to emphasize that the gut microbiota is disturbed in IBD. The presence of *Mycobacterium avium* subsp. *paratuberculosis* and adherent-invasive *Escherichia coli* is increased in the IBD patients, as well as *Clostridium difficile*, *Enterobacteriaceae*, *Bacteroides* and *Eubacteria*. Additionally, decreased amount of the anti-inflammatory commensal, e.g., *Faecalibacterium prausnitizii* is also found in IBD [40,41,42]. IBD patients also demonstrated higher levels of facultative anaerobes and lower levels of obligately anaerobic producers of SCFA [43]. Interestingly, recent data indicate that gut microbiota disorders are also associated with CRC. Several bacteria, such as *Fusobacterium nucleatum* and certain strains of *Escherichia coli* and *Bacteroides fragilis* may play a role in the carcinogenesis of CRC [44]. Disturbances of the composition of the gut microbiota may contribute to the fermentation of polyphenols, reducing their effectiveness. In contrast, polyphenols appear able to restore the content of gut microbiota in inter alia IBD, increasing the growth of the SCFA-producing bacteria exerting an anti-inflammatory effect [43,45]. It is difficult to confirm whether polyphenols are able to restore the proper composition of gut microbiota before their fermentation by bacteria; this may well depend on the doses of the compound. Nonetheless, further studies assessing the correlation between gut microbiota and polyphenols are needed to confirm this.

### 2.4. Bioavailability and Bioaccessibility of Polyphenols

Adequate bioavailability and bioaccessibility are key problems determining the usage and effectiveness of new agents, including polyphenols, against various diseases. The bioaccessibility of polyphenols is associated with the amount of compound accessible for absorption [46]. This quantity differs from the amount introduced by oral application due to inter alia instability during digestive processes (pH, digestive enzymes) and interaction with the food matrix, which disturbs the structure of the polyphenolic compounds and their properties. It must be emphasized that unmodified polyphenols should be delivered to the inflamed tissue before absorption to exert their fully expected, pro-health effect. This is determined by the localization of the pathological process. As both UC and CRC occur in the colon, the distal part of digestive tract, low amounts of polyphenols are delivered, resulting in low bioaccessibility; however, better bioaccessibility would be observed for diseases in the upper parts of digestive tract, as in CD (stomach, small intestine). In diseases with a more distal localization, the effect of the polyphenol depends on its bioavailability, which is associated with the serum level of polyphenols after absorption.

Unfortunately, polyphenols are characterized by low bioavailability, as determined by factors affecting bioaccessibility, the metabolic processes mediated by the liver (phase I and II metabolism), and the potential of vasodilatation to increase absorption and consequently serum level. Data suggest that the bioavailability of polyphenolic compounds decreases from phenolic acids to isoflavones, flavonols, catechins, flavanones, proanthocyanidins, and anthocyanins [47]. It is important to emphasize that polyphenols could be intrarectally administered, reducing the limitations associated with the oral application; however, oral drug delivery is one of the most convenient and preferred routes of administration because it is non-invasive, has high patient compliance, and is cost-effective. Moreover, self-administration through oral delivery is especially friendly to patients with chronic diseases that need frequent dosing [48,49]. Therefore, to achieve the desired effect of polyphenols in IBD and CRC, it is necessary to develop polyphenol encapsulations that can deliver compounds in non-changed form.

### 2.5. Appropriate Doses of Polyphenols and Side Effects Occurring after Their Usage

Some in vivo studies have reported that polyphenols have adverse effects such as iron deficiency, nephrotoxicity, and hepatotoxicity, particularly when high doses are used [50,51]. This may manifest as downregulation of antioxidant enzymes and heat-shock protein expression or enhanced production of malondialdehyde and 4-hydroxynonenal, i.e., harmful products of lipid peroxidation, in the liver and kidneys [52,53]. Additionally, it was demonstrated that polyphenols with neuroprotective properties blocking amyloid β aggregation have pro-oxidant activity [54]; this may contribute to DNA damage and the apoptosis of normal cells, as well as altered redox status of endothelial cells, inducing cardiovascular diseases [55,56]. On the other hand, polyphenols may interact with other drugs, changing their therapeutic concentration in the serum and hence their effects [57]. In contrast, low concentrations of polyphenols may have a protective outcome on hepatic and renal damage [58]. Hence, establishing the correct dose of polyphenols is difficult and requires special attention. Further studies are necessary to determine the profile of side effects occurring after supplementation of polyphenols and recommended doses with pro-health activity; these should also determine the toxicity of polyphenols alongside their therapeutic effects.

**Figure 1:**
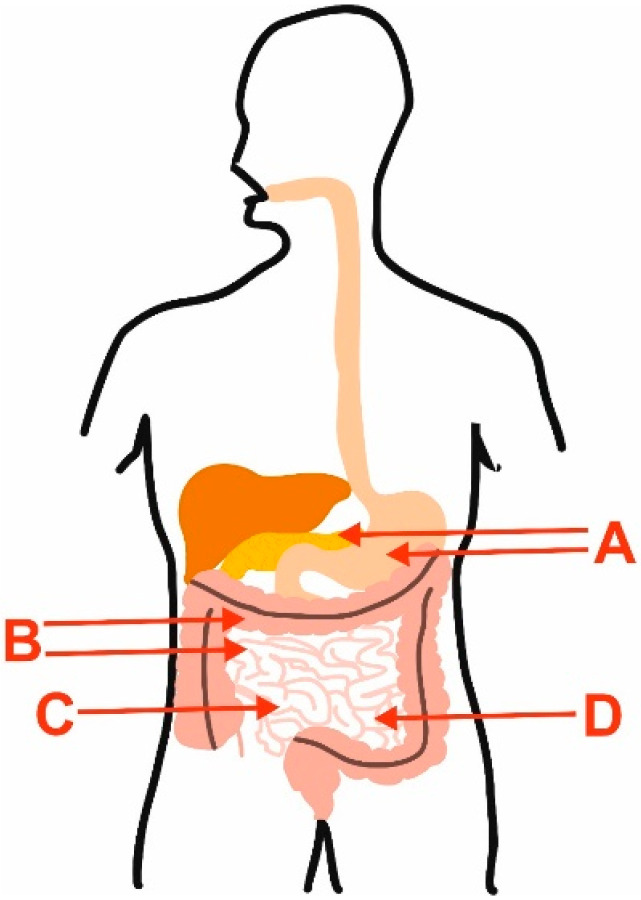
Main limitations of the use of polyphenols in IBD and CRC, which may be overcome by encapsulation of these compounds. A—digestive enzymes and pH; B—changed motility of intestine and colon; C—disruption of the intestinal barrier; D—metabolism of polyphenols by gut microbiota.

To enhance the poor bioaccessibility and stability of polyphenols, and to overcome the other limitations mentioned above, encapsulations may be used to reduce the need for high doses and hence their potential side effects (Figure 1). In addition, their use may also mask the flavor and control release of polyphenolic compound.

## 3. Encapsulations of Polyphenolic Compounds in IBD and CRC

Studies evaluating the efficacy and therapeutic value of polyphenol encapsulations in IBD and CRC are still in progress.

### 3.1. IBD

Novel bioformulations using polyphenols are being developed. These could represent an effective option for mitigating colitis and sustaining remission. Resveratrol-encapsulated microsponges delivered by pectin-based matrix tablets demonstrated better therapeutic effects than pure resveratrol in rats with colitis; treatment resulted in intact mucosal crypts and healthy mucosal and submucosal lines in the colon [59]. Silk fibroin nanoparticles, a vector for controlled release of resveratrol, reduced the macroscopic symptoms of inflammation and inflammatory markers and enhanced intestinal barrier function in an experimental model of IBD in rats; these results were better than for non-bioformulated polyphenol [60]. Pujara and co-workers showed that oral delivery of β-lactoglobulin nanospheres encapsulating resveratrol alleviated inflammation in Winnie mice with spontaneous UC [61].

One particularly interesting method uses nanoparticles formed of chitosan: a biocompatible and biodegradable polymer capable of mucosal adhesion and extending the retention time of the substrate. Nanoencapsulations could be used to more effectively target the inflamed colon in IBD; these include chitosan compositions obtained by ionic gelation of chitosan dispersions and resveratrol-loaded nanoparticles with tricarballylic acid, or a chitosan/alginate nano-drug delivery system loaded with resveratrol. Their efficacy would be enhanced via prolonged retention and delivery of the polyphenol [62,63].

Another promising treatment for UC is a colon-specific delivery formula of resveratrol targeting sphingosine kinase 1 (SphK1) and apoptosis. Administration was found to limit the inflammatory response in the colon and apoptosis of epithelial cells in rats with experimental UC [64]. Later, other agents were found to demonstrate effective suppression of colonic inflammation in IBD in vivo: rosmarinic acid-derived nanoparticles conjugated with poly(ethylene glycol), rosmarinic acid-loaded nanovesicles, oleuropein-loaded lipid nanocarriers, silica-installed redox nanoparticles with silymarin (compound being flavonolignan), or nanoparticles with curcumin [65,66,67,68,69].

Curcumin nanoparticles not only modulated the expression of the genes engaged in the inflammatory response in a rat model of UC; they also improved the mucosal lesions and preserved the distribution of telocytes, a distinct type of interstitial cells playing a vital role in colonic tissue homeostasis [70]. Interestingly, nanoparticles containing curcumin and catalase with poly(lactic-co-glycolic acid)-based surface functionalized with pluronic F127 enhanced mucus penetration and ROS-responsive drug release capacities for the delivery of curcumin to colitis tissues, thus resulting in more effective IBD therapy [71]. In addition, the efficacy of nanomicelles containing curcuminoids against UC symptoms was investigated in a randomized double-blind controlled trial; the study also included a self-reported measure of well-being [72].

Amyloid−epigallocatechin gallate (EGCG) hybrid nanofilament hydrogels demonstrate significantly higher polyphenol loading capacities, a long retention time in the colon, and very high stability. Oral administration strengthened intestinal barrier function, suppressed colonic inflammation. and regulated gut dysbiosis. The treatment also reduced the abundance of the operational taxonomic units associated with colitis, especially the facultative anaerobes of the phylum Proteobacteria, such as *Aestuariispira* and *Escherichia* [73].

Biodelivery strategies used for polyphenols could also increase the bioavailability of existing drugs used in the therapy of IBD. Tannic acid-based supramolecular nanoparticles offer promise for the oral delivery of anti-tumour necrosis factor alpha (TNF-α) antibodies in the treatment of experimental IBD; this approach is characterized by less systemic side effects due to enhanced accumulation in inflamed colon tissue [74].

### 3.2. CRC

Le and co-researchers demonstrated that tannic acid-containing nanoparticles, formed by a turbulent-mixing technique, exhibited uniform size, high stability, and pH-triggered drug release in the gastrointestinal tract, and could overcome intestinal mucosa for drug delivery in the colorectal region. This encapsulation was found to exert in vitro anti-inflammatory and antioxidant effects, through decreasing reactive oxygen species (ROS) and cytokine production, and was able to inhibit the development of inflammation-associated CRC. These nanoparticles also demonstrated anti-cancer activity showed by reducing tumour size in azoxymethane (AOM)/dextran sulphate sodium (DSS)-induced C57/BL6 mice after therapy. Moreover, this study confirmed that encapsulation in a polyphenol carrier improved therapeutic efficacy and provided a safe and effective nanoplatform for the polyphenolic compound [75].

One valuable encapsulation seems to be curcumin-poly (allyl amine) hydrochloride-based polymeric nanocapsules that can encapsulate curcumin with piperine, which acts as a glucuronidation inhibitor and enhancer of the curcumin bioavailability. The nanoencapsulation enhanced the physiochemical activities and solubility of curcumin, as well as its drug loading and release. In vitro and in vivo assays revealed that the curcumin nanocapsules caused selective and potential cytotoxic effects against colon cancer cells and diminished the protein expression of cyclooxygenase 2 (COX-2) and the activity of inducible nitric oxide synthase (iNOS) in vivo, inhibiting cancer cell proliferation and inflammation in carcinogen 1,2-dimethylhy-drazine (DMH)-induced CRC [76]. In turn, the encapsulation of curcumin by fusion protein GE11-HGFI can form uniform and stable nanoparticles; this formulation targeted CRC cells with high epidermal growth factor receptor expression, causing high aggregated concentrations of polyphenol at tumour sites, exerting a significant anticancer effect [77].

Some studies have assessed the therapeutic value of encapsulations with resveratrol. Encapsulating resveratrol in colloidal mesoporous silica nanoparticles (MCM-48-RES). This approach improved saturated solubility by about 95% and elevated in vitro release kinetics compared to pure resveratrol and caused a cytotoxic effect mediated via the poly ADP-ribose polymerase (PARP) and cellular inhibitor of apoptosis protein 1 (cIAP1) pathways in HT-29 and LS147T cells. In addition, the MCM-48-RES also inhibited the lipopolysaccharide (LPS)-stimulated inflammatory response in RAW264.7 cells by downregulating nuclear factor kappa B (NF-κB) pathway activation, suggesting that it may have therapeutic potential against IBD-associated CRC [78]. In contrast, co-encapsulating pristine resveratrol alongside cyclodextrin–resveratrol inclusion complexes in the lipophilic and hydrophilic compartments of liposomes resulted in complete (100%) drug release in 24 h and dose-dependent cytotoxicity thus exerting chemotherapeutic activity against HT-29 CRC cells [79]. Feng and co-workers found resveratrol-loaded lipid-core nanocapsules to have pro-apoptotic effects against HT-29 cells. The nanocapsules presented a typical endocytosis-mediated cellular internalization process and became located in the cell cytoplasm; they also demonstrated a controlled and sustained release pattern with a maximum release up to approximately 70% by the end of the 48-h study period [80]. The cytotoxicity, pro-apoptotic, and oxidant potentials of resveratrol were also found to be enhanced in CRC cells using zein nanoparticles, and significantly enhanced cellular uptake was detected compared to free resveratrol. Zein, which was utilized to the production of nanoparticles, is a biocompatible and inexpensive excipient used in the pharmaceutical industry and is obtained from maize plant [81].

On the other hand, the encapsulation of polyphenols may be used to enhance the effect of chemotherapeutics. Combinatorial treatment with oxaliplatin- and resveratrol-loaded N,O-carboxymethyl chitosan nanoparticles induced anti-cancer effects against CRC in vitro and in vivo, promoting apoptosis and decreasing the protein expression of α-SMA and CUGBP1 associated with fibrotic processes. Furthermore, the delivery of oxaliplatin and resveratrol by nanoparticles demonstrated stronger effects than the free drugs or either type of nanoparticles used alone [82].

It is worth emphasizing that the encapsulation of polyphenols may comprise two compounds. Kumar et al. evaluated the colon cancer targeting effectiveness of chitosan-coated-transresveratrol and ferulic acid-loaded solid lipid nanoparticles conjugated with folic acid in vitro. The encapsulates effectively involved and increased cytotoxicity in CRC cells and induced apoptosis more effectively than free resveratrol and ferulic acid. The action resulted from inter alia a reduction in the protein expression of cyclin B, D,1 E, and Cdk-2, -4, -6, factors engaged in cell cycle regulation [83].

Selected studies investigating the beneficial effects of individual encapsulated polyphenolic compounds in IBD and CRC are summarized in Table 1.

The above data demonstrate the potential of various encapsulations of polyphenolic compounds in IBD and CRC, including IBD-associated CRC. Nevertheless, clinical studies based on encapsulates with polyphenols are still lacking. This may result from insufficient clinical effectiveness. Therefore, there is still a need to identify new encapsulations of polyphenolic compounds.

## 4. Hemicelluloses as Compounds with Therapeutic Potential against IBD and CRC

### 4.1. Structure, Occurrence, Classes of Hemicelluloses

**Figure 2:**
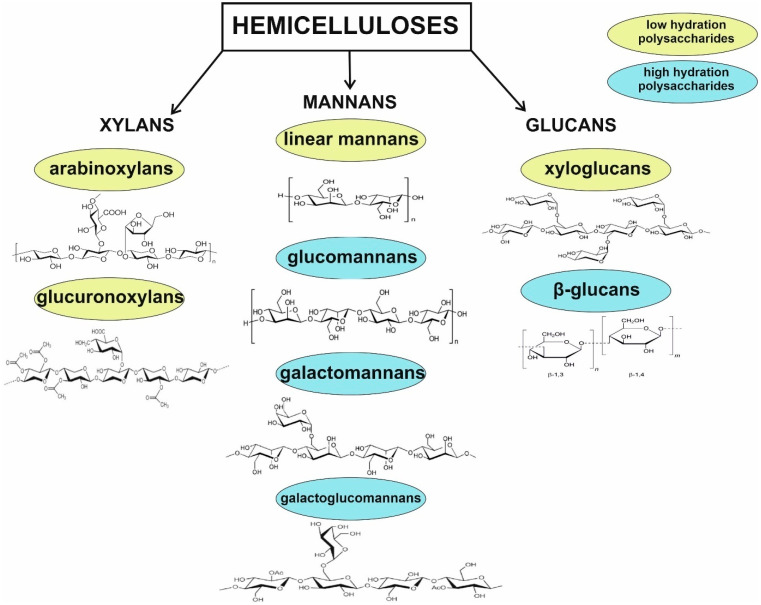
Division of common hemicelluloses. Glucomannans, galactomannans galactoglucomannans, and β-glucans belonging to high-hydration polysaccharides are used for extracellular energy and raw material storage, as well as water retention mechanism in seeds. In turn, arabinoxylans, glucuronoxylans, and xyloglucans stabilize the cell wall through hydrogen-bonding interactions with cellulose and covalent interactions with lignin. Due to their branched structure, they are water-soluble in their native state. Linear mannans are described as seed storage compounds.

Hemicelluloses are a diverse group of polysaccharides, which serve as components of plant cell walls. Although all are constructed from β-(1→4)-linked backbones with an equatorial configuration, they comprise three main classes: xylans, mannans, and β-(1→3,1→4)-glucans. Hemicelluloses are synthesized by glycosyltransferases located in the Golgi membranes. It is worth to emphasize that some studies classify galactans, arabinans, arabinogalactans, and callose as hemicelluloses; however, the first three compounds seem to be part of pectin molecules, representing a sidechain rather than a backbone, and their structures are not characterized by the equatorial β-(1→4)-linked backbone structure. In addition, galactans are synthesized by a distinct glycosyltransferase family that differs from xylans, mannans, and glucans. In turn, callose has a backbone entirely composed of β-(1→3) linked glucose residues. Therefore, these compounds should not be included as hemicelluloses [84,85,86]. Some subclasses of hemicelluloses, such as glucuronoxylans, arabinoxylans, linear mannans, glucomannans, galactomannans, galactoglucomannans, β-glucans, and xyloglucans, occur depending on plant developmental stage, tissue type, and species [87] and can vary in terms of the degree of hydration, function, and branched structure (Figure 2).

One of the most abundant groups of hemicelluloses present in residues produced by the agricultural industry are xylans, which may be extracted from plant biomass. Many food and agricultural products, such as wheat straw, corn stalks and cobs, sorghum and sugar cane, and hulls and husks from starch production, are sources of xylans [88]. It must be emphasized that xylan structure differs depending on the extraction method and compound origin. Typically, xylans from plants have a linear D-xylopyranose backbone linked by β-(1→4) glycosidic bonds. Depending on the source, the backbone may be partly acetylated or substituted with glucuronic acid, 4-*O*-methyl-glucuronic acid, and monosaccharides, including arabinose, xylose, and galactose, forming among other glucuronoxylans, arabinoxylans, glucuronoarabinoxylans, and other complex heteroxylans [88,89,90]. When using xylans as a material for polyphenol encapsulation, it is important to remember that the choice of raw source determines the efficiency and cost of extraction, the solubility of the obtained xylan, and its susceptibility to batch-to-batch variations. For example, the processes of extraction and purification of xylans from wood are costly. Additionally, wood-based xylans are characterized by limited solubility in water, restricting their use in synthesis of biomaterials [90]. Hence, the search for new sources of xylans with pro-health activities has aroused considerable interest [91,92,93].

Mannans are polymers of β-(1→4)-linked mannopyranosyl residues. Their structure may be linear or branched, the latter including glucose and galactose residues (glucomannans, galactomannans, and galactoglucomannans). A higher degree of backbone substitution is associated with greater solubility, and hence is more suitable for encapsulates [86]. Pure β-mannans are found in various natural sources, including palm seeds and algae. Glucomannans can be obtained from *Amorphophallus konjac* corms and orchid stems, galactomannans from legume seeds, and galactoglucomannans from gymnosperm stems (softwood) [94]. Mannans are used for carbohydrate storage in seeds and are present in the cell walls. They may also modify the mechanical properties of plant fibers or biocomposites by binding with cellulose bundles, thus providing resistance to mechanical damage. In the food industry, mannans are used as a stabilizer or thickening and gelling agent [95].

β-glucans are linear polymers linked to a D-glucose monomer by a β-1,3 or β-1,4 glycosidic bond; however, some have a branched structure containing β-1,6 linkages of varying lengths as well as cyclic structures (e.g., cyclic β-1,2-glucan). They are primary components of inter alia yeast, bacteria, seaweeds, oats, barley, and rye, with the species determining the configuration of the β-glucan; this can influence their molecular weight, solubility, type of glycoside bond connection, degree of branching, monosaccharide composition, and sugar chain conformation. The solubility of β-glucans determines their functional activity, and consequently, their application in the pharmaceutical and food industry. The compounds can be water-soluble or -insoluble; the former are characterized by lower toxicity, greater stability, and potential for the use as therapeutic agents [96,97]. Another glucan class comprises the xyloglucans, constituting up to 20–30% of all hemicellulose in the primary cell wall in dicots. The molecule consists of a β-1,4-linked glucan backbone partially substituted with xylosyl substituents at the O-6 position. It is important to note that xyloglucan has been widely studied due to its desired physicochemical properties, low processing cost, and broad regulatory acceptance [86,98].

### 4.2. Activity of Xylans against IBD and CRC

Xylans have a wide spectrum of pro-health activity. Arabinoxylan derived from finger millet mitigated hepatic inflammation in mice fed a high-fat diet; they also exerted anti-inflammatory effects by reducing the protein level of pro-inflammatory interleukin 1 beta (IL-1β), interleukin 6 (IL-6), and C-reactive protein (CRP) in the liver. In the same model, arabinoxylans prevented ileum damage and restored disturbed colonic barrier function, mainly by enhancing the expression of tight junction proteins, such as zonula occludens-1 (ZO-1), claudin 2, claudin 4, and occludin. In addition, oral supplementation modulated the composition of gut microbiota mitigating gut dysbiosis by decreasing the proportions of Firmicutes and Enterobacteriaceae and increasing that of commensal *Lactobacillus* spp., *Bifidobacterium* spp., and *Roseburia* [99]. In another obesity mouse model induced by a high-fat diet, arabinoxylan from rice bran reduced the serum level of LPS, IL-6, and TNF-α, markers of endotoxemia and inflammation, which are observed in IBD. Moreover, arabinoxylan from rice bran counteracted a decrease of the abundance of anti-inflammatory gut bacteria *Bifidobacterium* and *Akkermansia*, and a reduction of the level of anti-inflammatory butyrate in the colon, which also occurs in IBD [17,28].

MGN-3, an arabinoxylan from rice bran, was also found to reduce oxidative stress levels and inflammatory response indicators in mice: treatment resulted in greater superoxide dismutase (SOD), glutathione peroxidase (GSH-Px), and catalase (CAT) activity, and increased total antioxidant capacity in the serum, and in the jejunal and colonic mucosa. MGN-3 also reduced the genetic expression of pro-apoptotic caspase-3, 8, 9, and 10 and their enzymatic activities and upregulated the gene expression of tight junction proteins in the jejunal and colonic mucosa [100]. In addition, MGN-3 was found to increase the expression of nuclear factor erythroid 2-related factor 2 (Nrf2), thus preventing colon damage and the development of IBD-associated CRC [101,102]. As oxidative stress and reduced CAT, SOD, and GSH-Px activity are concomitant with enhanced apoptosis of epithelial colon cells and lowered expression of epithelial barrier proteins, both present during IBD, arabinoxylans derived from rice bran could improve intestinal and colonic barrier function.

Water-extractable high molecular weight arabinoxylans from wheat had prebiotic effects related to an increase in *Bifidobacteria* and *Roseburia* in the gut [103]. In contrast, short-chain arabinoxylans prepared from enzymatically treated wheat grain altered the microbiota composition towards butyrate producers in the caecum of broilers, reducing colonic inflammation by decreasing T-lymphocyte infiltration [104]. Wheat-derived arabinoxylans are also able to reduce the functional activity of M2-macrophages, which play a key role in maintaining intestinal homeostasis [105]. Furthermore, these compounds were found to act as immunomodulators of the inflammatory response in LPS-induced RAW264.7 macrophages; this was believed to act by suppressing the nitric oxide (NO) production that positively correlates with the increased pro-inflammatory cytokine levels characteristic of IBD [106,107].

Interestingly, xylans were also found to demonstrate positive effects in an animal model of IBD. Oral administration of a derivative of butyrate-releasing linear xylan extracted from corn cobs using alkali-solution extraction and alcohol precipitation reduced inflammation in C57BL/6 mice with DSS-induced murine colitis; treatment reversed the imbalance between pro- (IL-1β, IL-17, TNF-α) and anti-inflammatory cytokines (IL-10) caused by DSS and rebalanced the composition of gut microbiota, reducing the relative abundance of the genera *Oscillibacter*, *Ruminococcaceae UCG-009*, *Erysipelatoclostridium,* and *Defluviitaleaceae UCG-01*. It also raised the butyrate content in the colon, upregulated G-protein-coupled receptor 109A protein expression, inhibited histone deacetylase (HDAC) activity, mitigating the course of the disease, and exerted anti-inflammatory activity by activating the autophagy pathway and inhibiting the nuclear factor kappa B (NF-κB) pathway. These changes led to the suppression of inflammatory intestinal damage, indicating that the agent had potential for treating UC [108].

It is known that the presence of chronic inflammatory conditions in the colon, such as IBD, increases the risk of CRC [109]. Xylans may exert a chemopreventive effect in IBD by limiting inflammation and oxidative stress and may possess anti-cancer potential. For instance, BioBran/MGN-3 standardized arabinoxylan is described as a complementary compound for conventional cancer treatment. It is able to exert immunomodulatory, pro-apoptotic anti-cancer effect, mainly by stimulating natural killer (NK) cells, upregulating p53 expression and increasing Bax/Bcl2 ratio. MGN-3 may induce damage to DNA, increase the susceptibility of cancer cells to chemotherapeutics and enhance phagocytosis [110,111]. Additionally, the addition of arabinoxylans derived from rice bran to the curcumin therapy may limit the progression of cancer [112].

The biological activity of xylans, mainly arabinoxylans, depends on their chemical structure; for example, the arabinose of arabinoxylan may be bound to some compounds, such as phenols, and hydrolysates of the arabinoxylans may exert stronger anti-cancer effects than non-modified compounds. They are able to alleviate Caco-2 cell barrier damage by regulating the TLRs/MyD88/NF-κB pathway, decreasing pro-inflammatory factor (IL-8, TNF-α) production in colon cancer cells (Caco-2 and HT-29), and inhibiting the viability of HCT-116 cells [113,114,115]. Nevertheless, as arabinoxylan hydrolysates form after enzymatic modification of arabinoxylan, these compounds will not be exhaustively covered in this review.

### 4.3. Activity of Mannans against IBD and CRC

Mannans are able to inhibit the inflammatory response in macrophages and appear to play an important role in the development, propagation, control, and resolution of IBD; mannans from cabernet franc, cabernet sauvignon, and sauvignon blanc wines were found to reduce the production of pro-inflammatory factors (IL-1β, TNF-α, NO) in LPS-induced RAW264.7 cells [116]. Additionally, two glucomannans from *Bletilla formosana* (BFP60 and BFP80) downregulated the production of pro-inflammatory cytokines (IL-1β, IL-6, TNF-α) and enzymes (COX-2, iNOS) by suppressing the NF-κB pathway in an in vitro mouse macrophage model [117].

In addition, β-galactomannan inhibited the mRNA expression of IL-1α, IL-6, TNF-α, granulocyte-macrophage colony-stimulating factor (GM-CSF), and chemokines CCL2, CCL20, and CXCL8, as well as the protein expression of IL-6 and CXCL8, in *Salmonella*-induced ileum intestinal epithelial cells in vitro, which replicated the intestinal inflammatory response [118]. Additionally, mannan obtained from yeast cell walls alleviated deoxynivalenol-induced injury in jejunum epithelial cells; it also counteracted the down-regulation of intracellular reduced glutathione (GSH) and the up-regulation of reactive oxygen species (ROS), malondialdehyde (MDA), and pro-inflammatory IL-6, IL-8, and TNF-α. It also suppressed cell apoptosis by activating the PI3K-AKT-mTOR signaling pathway, thus inhibiting autophagy, and alleviating intestinal damage [119,120].

In turn, galactoglucomannan was found to mitigate chronic inflammation of prostate by reducing the abundance of harmful Odoribacter and Clostridiaceae in the gut and increasing the level of SCFA in feces [26]. This suggests that galactoglucomannan may be able to modify the composition of gut microbiota and enhance SCFA production, exerting desirable effects in IBD and CRC. Additionally, other mannans, such as Konjac glucomannans or yeast mannans, may regulate the composition of gut microbiota, exerting pro-health effect by a decreasing the abundance of harmful bacteria and promoting the growth of *Lactobacillus* strains that can improve the environment of the intestinal tract [121,122].

Mannans appear to have strong antioxidant potential. For instance, galactomannans extracted from Chinese *Sesbania cannabina* may increase the level of superoxide dismutase (SOD) and limit the production of intracellular ROS [123,124]. Other studies indicate that galactomannans deriving from *Delonix regia* and locust bean gum may have pro-healing properties [125,126]; this would be desirable for intestinal mucosal healing, regeneration, and improving intestinal barrier integrity in IBD. The galactomannan of *Delonix regia* seeds is able to limit inflammatory cell infiltration and increase fibroplasia and collagenesis by enhancing transforming growth factor beta (TGF-β) and alpha smooth muscle actin (α-SMA) protein expression in wounds. It may also suppress oxidative stress by increasing GSH and decreasing MDA [126]. Additionally, glucomannan from *aloe vera* gel was found to support intestinal stem cell-mediated epithelial regeneration via the Wnt/β-catenin pathway regulating intestinal homeostasis, thus regenerating the damaged intestinal barrier in IBD [127].

Mannans may also act as anti-cancer agents against CRC. Branched mannans from the mushroom *Cantharellus cibarius* were found to enhance the anticancer activity of NK cells against human colon cells and limit their growth by interfering with signal transduction in the NF-ĸB pathway [128,129]. Another acetylated mannan isolated from *aloe vera*-induced apoptosis in CRC cells via the mitochondrial pathway, altering mitochondrial membrane permeability by promoting Bax translocation; it also induced cytochrome-c release, initiating the caspase cascade reaction [130]. In contrast, aloe gel glucomannan caused colon cancer cell death through the mitochondrial damage-driven PINK1/Parkin mitophagy pathway [131].

### 4.4. Activity of Glucans against IBD and CRC

β-glucans derived from inter alia yeast, oats, barley, seaweeds, and mushrooms were found to have strong pro-health potential against IBD and CRC. They have a significant impact on the composition of the gut microbiota and in turn on IBD and CRC: treatment supports the growth of *Lactobacillus*, *Bifidobacterium*, *Rosebruia,* and *Ruminococcus*. In addition, they promote the formation of anti-inflammatory SCFA and exert immunomodulatory activities by influencing the migration and adhesion of immune cells and the expression of cytokines and by modulating cell proliferation and apoptosis. β-glucans exert chemopreventive properties against CRC by restricting inflammatory cytokine production, leukocyte activation, and metastasis. Moreover, they are able to inhibit tumor growth factors and exert a significant cytotoxic effect on cancer cells [132,133]. Studies on in vivo models of IBD caused by DSS-/2,4,6-Trinitrobenzene sulfonic acid (TNBS) have found oat and microalgal β-glucans to be effective at mitigating colitis. They appear to act by modulating the expression of chemokines, their receptors and tight junction proteins, as well as restoring gut microbiota composition and normalizing disturbances in the oxidative balance associated with inflammation of colon mucosa and submucosa [134,135,136,137]. Additionally, β-glucans may alleviate mitochondrial dysfunction in colitis [138]. In addition, 1,6-β-glucan and 1,3-β-glucan may downregulate autoimmune inflammation occurring in IBD [139]. Interestingly, (1→3)(1→6)-β-d-glucan has been found to have anti-nociceptive potential, suggesting that it could reduce abdominal pain occurring in IBD or CRC [140].

## 5. Methods of the Hemicellulose Preparation for Creation of Encapsulates and Types of Formulation

There exist different available methods for the extraction of hemicelluloses and their separation from remaining components of plant. Nevertheless, the most significant are techniques facilitating recovery of hemicelluloses into liquid phase [141,142]. However, actually the most popular are water extraction (subcritical water, autohydrolysis), dilute-acid extraction, alkali treatment, and organic solvent treatments. Each of them has its disadvantages. Nonetheless, the extraction using acids is characterized by the highest extraction yield [142]. Autohydrolysis is a process associated with pre-treatment of biomass using hot water and leads to the extraction of hemicelluloses into the liquid phase. In addition, it enables the hemicelluloses valuation [143]. It is worthwhile to emphasize that high temperature and pressure operational conditions are key factors in hydrothermal processes. They contribute to the water autoionization and disintegration of the plant biomass, as well as promote hemicellulose depolymerization [141,144]. Moreover, hydrothermal extraction is advantageous in terms of the simplicity of the device employed and the usage of non-toxic substances; however, the low extraction efficiency and a long, extended reaction time limit the use of this type of extraction [142,145]. In contrast, the main advantage of dilute-acid extraction over autohydrolysis is faster recovery of hemicelluloses compared to a simple autohydrolysis [146,147]. Nevertheless, the major disadvantage of dilute-acid extraction is difficulty in the separation of hemicellulose in hydrolysate [148]. The use of organic solvent may result in obtaining high-purity hemicellulose. Nonetheless, the common application of solvents, such as dimethyl sulfoxide and dioxane, is often associated with environmental pollution [142]. It has been demonstrated that hemicelluloses may be obtained with a high yield and high molecular weight using alkali treatments at a low temperature and pressure. The main limitations of this method are difficulties in the separation and purification of hemicellulose [142]. There are a lot of types of formulation based on hemicelluloses. They can enhance the therapeutic efficacy of the drug (polyphenols) and reduce the adverse effect and toxic reactions. Looking at the most popular formulations, we can describe them as follows. Nanoparticles are considered as safe matrices due to their capacity for easy modification. Generally, they are solid colloidal particles consisting of molecules ranging in size from 10 nm to 1000 nm. However, the size is usually 100–200 nm in nanomedicine. Nanoparticles have significant advantages to carry drugs that are typically dissolved and encapsulated. Liposomes are self-assembled bilayer vesicles having significant potential for use as carriers of many drugs. Due to their low permeability, drug molecules are usually encapsulated either in the hydrophilic core or the hydrophobic lipid bilayer. Polymeric micelles are widely used in drug delivery fields due to improvement of pharmacokinetics and biodistribution of drugs. The core of micelles may contain hydrophobic drugs, and the hydrophilic shell makes the micelle water soluble, facilitating delivery of the poorly soluble contents. Hydrogels are characterized by high water content and soft networks that may minimize the tissue irritation or cell adherence. Their porous structure and high water content cause that active substances can be encapsulated in hydrogels. Emulsions are very functional systems for encapsulating drugs dissolved in the disperse phase. They are colloidal mini-sized cargos, comprising two or more immiscible phases such as oil, water, and emulsifier (hemicellulose) [29,149].

## 6. Carriers and Encapsulates Based on Hemicelluloses

Data indicate that hemicelluloses, xylans, mannans, and glucans, are bioactive compounds. These have hence been labelled green renewable biopolymers, which are valuable agents for creating carriers and encapsulates, and may be useful in drug delivery systems [98,150,151]. Generally speaking, polyphenols easily interact with hemicelluloses, making them useful in polyphenol encapsulates; in addition, hemicelluloses may play a protective role, slowing the release of polyphenols in the human digestive system [152]. In addition, as hemicelluloses also inhibit the development and progression of IBD and CRC, encapsulation of polyphenols may result in synergistic activity and stronger effects against IBD and CRC compared to the individual polyphenols or formulations with other types of encapsulation. Moreover, hemicellulose encapsulation of polyphenolic compounds could also increase the anti-inflammatory, anti-oxidative, or anti-cancer potential and restore the composition of the gut microbiota, an etiological factor in IBD or CRC [17,153].

Studies evaluating the efficacy and therapeutic value of carriers and encapsulations based on hemicelluloses are ongoing. However, few studies have assessed the utility and pro-health potential of hemicelluloses encapsulation of polyphenolic compounds against IBD and CRC. The issue is still developing.

### 6.1. Xylan-Based Carriers and Encapsulates

Xylans, the predominant main representatives of hemicellulose, have strong potential for supporting the delivery of polyphenols by encapsulation. For instance, xylan nanoparticles are characterized by high resistance to breaking, and are biodegradable, biocompatible, non-toxic, and non-immunogenic, making them suitable for IBD and CRC therapy. They are typically synthesized by precipitation and dialysis [150,154,155]. It is important to note that the preparation of high-quality xylan-based encapsulates is a complex procedure due to the need for xylan to be purified from other hemicellulose-derived monomers and the creation of special functionalized linkages [150]. Data indicate that xylan-based nanoparticles are typically bound to the delivered agent (polyphenol) through ester (covalent) linkages, while other nanoparticles tend to be bound through non-covalent linkages and other hydrophobic interactions. Hence, xylan-based encapsulates could be more resistant to the acidic environment of stomach and early degradation. In addition, as they can only be degraded by enzymes produced by the colon microbiota, such as β-glucuronidase, α-arabinosidase, and β-galactosidase, xylan-based encapsulates may serve as colon-specific drug carriers. Xylan-based encapsulates have previously been used to deliver drugs against IBD and CRC, such as 5-fluorouracil (5-FU) or 5-aminosalicylic acid (5-ASA, mesalamine), suggesting they may also demonstrate potential for the delivery of polyphenols in these diseases [154,156]. One study examined drug release from gastro-resistant capsules filled with mesalamine-loaded xylan microparticles produced by cross-linking polymerization with a non-hazardous cross-linking agent. The xylans were extracted from corn cobs. It was found that this encapsulation had better control of the drug during different simulated gastrointestinal media in vitro and that a significant amount of the drug may be able to reach the large intestine and exert a stronger therapeutic effect in IBD [157]. Additionally, xylans isolated from corn cobs were used in the synthesis of xylan–stearic acid conjugate-based nanoparticles delivering 5-FU. The encapsulates induced higher cytotoxicity against human colorectal cancer cells (HT-29 and HCT-15) compared to the free drug. These results suggest that this may be a promising delivery system that could be used for polyphenol encapsulation in treating CRC [158].

A literature review revealed only a few studies whose aim was to synthesize and assess the biological activity of polyphenols encapsulated with xylans. Kowalska and co-researchers created arabinoxylan-based microcapsules loaded with honey polyphenols. The arabinoxylans were water-soluble hemicelluloses composed of a β-(1–4)-xylopyranose linear backbone chain with α-L-arabinose substitution at O-3 and/or O-2 position, derived from rye bran. It should be emphasized that ferulic acid molecules may be ester-linked to some arabinose residues at O-5 position of arabinoxylan, facilitating the formation of dimers, trimers, or even polymers. These can serve as the primary component of a carrier and improve its physicochemical properties; as such, carriers formed from arabinoxylans are stable to pH and temperature, are suitable as matrices used for controlled release delivery systems of bioactive ingredients, and can absorb high amounts of water. They are also degraded by the colonic microbiota, causing the release of their payload in the intestine. In addition, these compounds have prebiotic properties and may be fermented in the colon to SCFA, stimulating the growth of lactic acid bacteria. The honey polyphenols were encapsulated by spray drying at an inlet/outlet temperature of 110/65 °C being the most commonly used encapsulation method, due to the relatively low cost of the process, the availability of equipment, and the low heat load of the carrier material; it can also be used with substances sensitive to high temperatures. The arabinoxylan-based encapsulation enhanced the antioxidant activity of the honey polyphenol by 52%, 55%, and 471%, according to the DPPH, ABTS+, and FRAP assays measuring the activity of antioxidants, respectively [159].

Arabinoxylan-based microcapsules loaded with honey polyphenols also effectively inhibited the inflammatory response in LPS-stimulated RAW 264.7 macrophages by lowering IL-6 and TNF-α secretion, and NO production. In addition, simulated gastrointestinal digestion found that encapsulation had a protective role for polyphenols, resulting in higher levels of the polyphenolic compound in both the small and large intestine, highlighting the potential of these encapsulates in IBD therapy [160]. As macrophages play an important role in the development, propagation, control, and resolution of IBD [161], limiting the inflammatory response mediated by macrophages may lead to remission.

Sauraj and co-researchers created two types of nanoparticles based on xylans from corn cobs, which improved and enhanced therapeutic efficacy of curcumin in CRC treatment. The first type consisted of pH-responsive nanoparticles developed by directly conjugating the curcumin to the xylan backbone via an acid labile succinate linkage. The synthesis of biomaterial was confirmed through FT-IR, ^1^H NMR, UV–vis, and fluorescence spectroscopy. The tested nanoparticles were characterized by significantly faster drug release at a mildly acidic pH of 5.0 than a physiological pH of 7.4; this is a favorable characteristic when targeting CRC as a pH of about 5.0 is characteristic of tumour microenvironments, especially in the colon. In addition, it was revealed that nanoparticles could efficiently deliver curcumin to the nucleus of the tumour cells and exert much more cytotoxic effects against HT-29 and HCT-15 CRC cell lines than curcumin alone [162]. The second type of xylan-5-FU-curcumin nanoparticles also demonstrated anti-cancer activity against CRC cells; this was stronger than that of free curcumin and 5-FU, inducing enhanced cell apoptosis. Redox-responsive xylan–curcumin nanoparticles were characterized by high curcumin loading and stability and showed excellent redox-responsiveness due to the disulfide linkage and curcumin release [163]. Both types of xylan–curcumin nanoparticles offer promise as polyphenol encapsulates, effectively delivering curcumin to CRC cells. In addition, resveratrol encapsulated in bagasse xylan/resveratrol graft-esterified nanoparticles demonstrated anti-cancer potential that could be used in CRC [164].

Encapsulating polyphenols in hydrogels also offers promise. Gami et al. synthesized novel hydrogels from xylan (derived from corn) and β-cyclodextrin loaded in curcumin and 5-FU using ethylene glycol diglycidyl ether as a crosslinker in alkaline medium at different molar ratios. The physical and chemical properties of the encapsulates were determined by a swelling study and Fourier transform infrared spectroscopy, respectively. The morphological analysis demonstrated the porous structure of hydrogels, and the rheological study showed the flow behavior and gelation characteristics of the encapsulates. It was found that the drug release kinetics were best fitted by Korsmeyer–Peppas model. From the kinetic model fitting, it may be concluded that in vitro release of curcumin from xylan-β-cyclodextrin hydrogel follows three stages, and the highest cumulative release of curcumin was after 24 h [165]. These results suggest that the tested hydrogel has the capability to encapsulate polyphenol and could be used as delivery material against CRC and other cancers. However, further studies are needed to confirm its pro-health potential.

Another study examined the encapsulation of gallic acid in hydrogels based on arabinoglucuronoxylan from wheat bran, both in situ and ex situ. It compared two types of arabinoglucuronoxylan micro-hydrogel encapsulates, produced enzymatically using recombinant α-L-arabinofuranosidase, which selectively removes arabinose side chains and those formed by coacervation. The enzymatically produced hydrogels were characterized by higher zeta potential (−8.8 mV) and retained and released gallic acid with higher antioxidant capacity than chemically formed micro-hydrogels; however, the chemically produced micro-hydrogels demonstrated greater polyphenol encapsulation (72% in situ vs. 68% ex situ) than the enzymatically formed micro-hydrogels (59% in situ vs. 52% ex situ). Moreover, enzymatic modification and in situ encapsulation were the most effective methods for producing xylan-based encapsulation of gallic acid, allowing the release of a functional payload [166]. This encapsulate, in the form of a hydrogel, could be a valuable candidate for medical application supporting therapy of diseases.

The literature review also revealed other xylan-based carriers, tested mainly in vitro, which could be used to improve the therapeutic effectiveness of polyphenols in IBD and CRC. Mendez-Encinas and co-researchers prepared arabinoxylans-based particles by coaxial electrospraying. The arabinoxylan-based gels presented a spherical shape and rough surface with a three-dimensional and porous network. They did not induce proliferation of human normal colon epithelial cells (CCD 841 CoN) nor any toxic effect on them, suggesting that they are safe for use in creating encapsulates of compounds, e.g., polyphenols, with pro-health activities in colon diseases [167].

Another study looked at core-shell hybrid nanoparticles formed by a silica core and xylan carrying a 5-(4-hydroxyphenyl)-10,15,20-triphenylporphyrin (TPPOH) shell; the TPPOH shell was covalently bound to xylan. The nanoparticles were found to improve drug-controlled incorporation and blood circulation. The xylan-TPPOH-coated nanoparticles demonstrated about 40-fold greater effectiveness against HCT-116 cells compared to free TPPOH, and 10-fold greater effectiveness against HT-29 colon cancer cells [168]. They also limited the tumor volume in mice in vivo [169]. Hence, xylan-coated nanoparticles are promising agent carriers in the treatment of CRC.

Other studies focused on xylan/andrographolide grafted and esterified derivative nanoparticles or xylan/andrographolide folate-g-dimethylaminoethyl methacrylate/diethylene glycol dimethacrylate nanoparticles; the xylans were derived from bagasse. Their structure and properties were characterized by FTIR, XRD, DTG, SEM, and ^1^H NMR assays. The carriers demonstrated anti-cancer activity [170,171].

Microparticles based on corn cob-derived xylans were prepared by crosslinking polymerization using sodium trimetaphosphate. The microparticles were assessed for morphology, particle size, polymer-cross-link agent interaction, and in vitro toxicity. FT-IR analyses revealed an interaction between sodium trimetaphosphate and xylan during the cross-linking process with the formation of phosphate ester bonds. Furthermore, X-ray diffraction patterns and FT-IR analyses indicated that little or no cross-linking agent remained inside the microparticles. In addition, in vitro assays using human erythrocytes showed that the microparticles are not toxic. Hence, these xylan microparticles may be used as a platform for creating encapsulates of compounds with therapeutic potential against diseases [172].

### 6.2. Mannan-Based Carriers and Encapsulates

Another valuable base material for carriers, which subsequently could be used for the formation of encapsulates of polyphenols, are mannans, particularly galactomannans [173].

Our literature review revealed some novel encapsulations of curcumin based on konjac glucomannans. Meng and co-researchers prepared curcumin-loaded konjac glucomannan octenyl succinate (CKGOS) nanoemulsion with a high loading capacity. CKGOS was used as the wall material of the encapsulate. These particles were characterized by spherical self-aggregating morphology with a rough matte edge, as well as good thermal processing and storage stability. In vitro and in vivo stability assays suggested that tested CKGOS nanoemulsion gave good protection of curcumin and enabled colon-targeted delivery of polyphenol, improving its bioaccessibility. In vivo gastrointestinal propulsion revealed that a much higher amount of curcumin reached the colon from the encapsulates compared to free curcumin [174]. It demonstrates that these encapsulations could be applied to support the treatment of colon diseases.

In turn, Wang and colleagues entrapped curcumin in multilayered emulsions to increase its stability and bioavailability. The encapsulation was stabilized by whey protein isolate and coated with carboxymethyl konjac glucomannan. The curcumin-loaded emulsions were stable with a narrow size distribution and were generated by layer-by-layer assembly according to confocal laser scanning microscope observation. Interestingly, the carboxymethyl konjac glucomannan located at the outermost layer of the encapsulates slowed the release of curcumin in simulated gastric fluid. However, enhanced release of curcumin was found from encapsulates in the simulated colonic fluid containing β-mannanase. In addition, in vivo experiments using mice showed that the bioavailability of curcumin contained in the encapsulates was increased by approximately four-fold compared to curcumin alone, indicating that these multilayered emulsions coated with mannan may be promising vehicles for colon-delivery systems in colon diseases [175]. Additionally, mannans may be used as one of the components in the carrier. For instance, a complex with carboxymethyl konjac glucomannan and chitosan was used to synthesize nanogels with 1-ethyl-3-(3-dimethylaminopropyl)/N-hydroxysuccinimide) (EDC/NHS)-initiated crosslinking. The resulting gels allowed a controlled and pH-dependent release profile of curcumin in simulated gastrointestinal conditions, as well as high curcumin encapsulation [176].

Konjac glucomannan also was used to encapsulate anthocyanins: another set of polyphenols with anti-inflammatory, anti-oxidative, and anti-cancer activities. Nguyen and co-researchers compared microencapsulations of these compounds using various chemicals, such as konjac glucomannan, maltodextrin, gum arabic, or inulin, through spray-drying and freeze-drying techniques. Among the tested encapsulates, konjac glucomannan had the highest phenolic, anthocyanin, and antioxidant activity when used with anthocyanins from hibiscus (*Hibiscus sabdariffa* L.) calyces. Interestingly, the microcapsules prepared by spray-drying displayed higher antioxidant potential than these formed by freeze drying. However, this type of encapsulate was characterized by the lowest encapsulation efficiency [177].

Guar gum, a galactomannan obtained from the seeds of *Cyamopsis tetragonolobus*, was found to be adequate material for the synthesis of anthocyanin encapsulates. Paula and co-workers showed that guar gum increased the thermal stability of anthocyanins at pH 4.0 in aqueous dispersions and in double emulsions W/O/W. It also improved the total final anthocyanin content, demonstrating a 2.4-fold increase in the half-life time of anthocyanins, and increased the antioxidant capacity of the sample. The double emulsion demonstrated high encapsulation efficiency for the polyphenols (90.6%) and high kinetic stability, and protected the anthocyanin molecules against degradation, suggesting their potential for treating colon diseases [178]. Additionally, in microcapsules with anthocyanin-rich aqueous chokeberry (*Aronia melanocarpa*) fruit extract or microcapsules with grape (*Vitis labrusca* var. Bordo) skin phenolic extract, guar gum encapsulations were found to be effective at maintaining the stability of polyphenols, which may be valuable in treating IBD and CRC [179,180].

### 6.3. Glucan-Based Carriers and Encapsulates

In addition to xylans and mannans, glucans also seem to be suitable material for creating carriers used for encapsulating polyphenols [181]. Encapsulates based on oat β-glucan, such as micelles or nanocapsules, appear to be suitable for delivering curcumin to the colon [182,183]. For instance, Liu et al. investigated the stability of curcumin-loaded nanocapsules composed with self-aggregates of octenylsuccinate oat β-glucan over storage and digestive fluids. The encapsulates were found to demonstrate high stability during storage and thermal stimulation, which was better than free curcumin. Additionally, the nanocapsules were more stable than the polyphenol alone in mimetic intestinal fluids, indicating that the carrier offers effective protection for curcumin in digestive environments. Moreover, the simulated passage through mimetic gastric and intestinal fluids revealed that curcumin was tightly accommodated in the capsule, but rapidly escaped as the capsule reached the colon, thus favoring higher bioaccessibility [183].

Interestingly, some in vitro and in vivo studies have examined the potential of curcumin encapsulation in yeast glucan particles against IBD. Plavcová and co-researchers created particles based on β-1,3-glucan from instant baker’s yeast, *Saccharomyces cerevisiae*, which were used to encapsulate curcumin by controlled evaporation of the organic solvent by the slurry evaporation method. The tested agents yielded anti-inflammatory effects in LPS-stimulated THP1-XBlue™-MD2-CD14 human monocyte cells. The particles decreased the secretion of IL-1β and TNF-α and reduced the activity of NF-κB/AP1 significantly more effectively than curcumin alone, demonstrating the anti-inflammatory potential of the encapsulates [184]. Additionally, another set of glucan particles consisting of β-1,3/1,6-glucans from *Saccharomyces cerevisiae*, synthesized by the same method, demonstrated a therapeutic effect against IBD. The administration of composites of yeast glucan particles with curcumin by gastric gavage was found to lower symptoms of colitis in DSS-induced Wistar rats. The encapsulates significantly lowered disease activity index, as well as the protein expression of pro-inflammatory IL-1β, IL-6, and TNF-α in the colon. These effects were greater compared to those of non-encapsulated curcumin [185]. Singh et al. found curcumin loaded into chitin–glucan quercetin conjugate to have anti-cancer potential. This combination was found to be cytotoxic in a macrophage cancer cell line (J774) and to have antioxidant activity, revealed by DPPH and ABTS^+^ radical-scavenging activity assays, indicating that these agents have potential against IBD-associated CRC [186].

Encapsulates of other polyphenols have also demonstrated anti-inflammatory properties that could be used in mitigating IBD. Yeast glucan particles incorporated with EGCG, a polyphenol extracted from green tea, or trans-resveratrol prepared by slurry evaporation and spray drying, were able to exert anti-inflammatory and antioxidant effects; however, greater activity was observed for the spray-dried encapsulates. EGCG/resveratrol loaded into glucan carriers showed greater anti-inflammatory potential in vitro than a simple suspension of EGCG/resveratrol, as demonstrated by inhibition of NF-κB/AP-1 and pro-inflammatory TNF-α secretion in LPS-induced THP1-XBlue™-MD2-CD14 monocytes [187].

EGCG was also used to support berberine, another natural compound, to designing a safe oral drug delivery system targeting macrophages. Both compounds were encapsulated into yeast microcapsules. In an in vivo model of UC, i.e., DSS-stimulated C57BL/6 mice, encapsulation transformed M1 macrophages into anti-inflammatory M2 macrophages, exerting specific anti-inflammatory effects. This was attributed to the interaction between β-1,3-D-glucan on the surface of the microcapsules and dectin-1 receptors on the macrophages. Furthermore, the released agents, primarily EGCG, decreased the levels of IL-1β and TNF-α in the colon tissue, increased colon length, prevented body weight loss, and mitigated histological colon damage [188].

Ahmad and co-researchers compared the potential of β-glucan isolated from barley (*Hordeum Vulgarea*) and β-cyclodextrin for spray-drying encapsulation of saffron (*Crocus sativus* L.) anthocyanins. They revealed that the powder yield and encapsulation efficiency of powders with β-cyclodextrin increased approximately 5% and 18%, respectively, compared to the powders with β-glucan. Nevertheless, the release of anthocyanins was more controlled from the microparticles based on β-glucan in simulated intestinal fluid and the gastric system. In addition, this encapsulation prevented the loss of polyphenolic compounds in the harsh conditions of the stomach. Hence, β-glucan could be an efficient and effective material for improving the bioaccessibility and bioavailability of polyphenols, as efficient encapsulation yielded a high retention of the core material retention inside the wall material [189].

Selected studies investigating the carriers and encapsulates based on hemicelluloses are summarized in Table 2.

## 7. Conclusions

There are strong limitations for the use of polyphenols in the treatment of IBD and CRC, which can be addressed by creating encapsulates. However, adequate encapsulates of polyphenol for use in IBD and CRC therapy remain unknown, and research into synthesizing polyphenol encapsulates and assessing their utility in IBD and CRC continue. Suitable biomaterials for carriers of polyphenol encapsulates are sought. One promising group of candidates are the hemicelluloses; in addition to their low toxicity, they demonstrate prebiotic, anti-inflammatory, anti-oxidative, and anti-cancer properties that could prevent the occurrence of CRC and induce and maintain the remission of IBD. Therefore, creating encapsulates of polyphenols with hemicellulose carriers would improve efficacy of polyphenols against IBD and CRC as well as increase their bioaccessibility and bioavailability. Hemicelluloses contained in encapsulates, on the other hand, released in bowels and having pro-health activities could intensify action of polyphenols against IBD and CRC. However, further studies are necessary to determinate detailed kind of the action effect of both compounds (synergistic/additive/based on mechanism).

**Figure 3:**
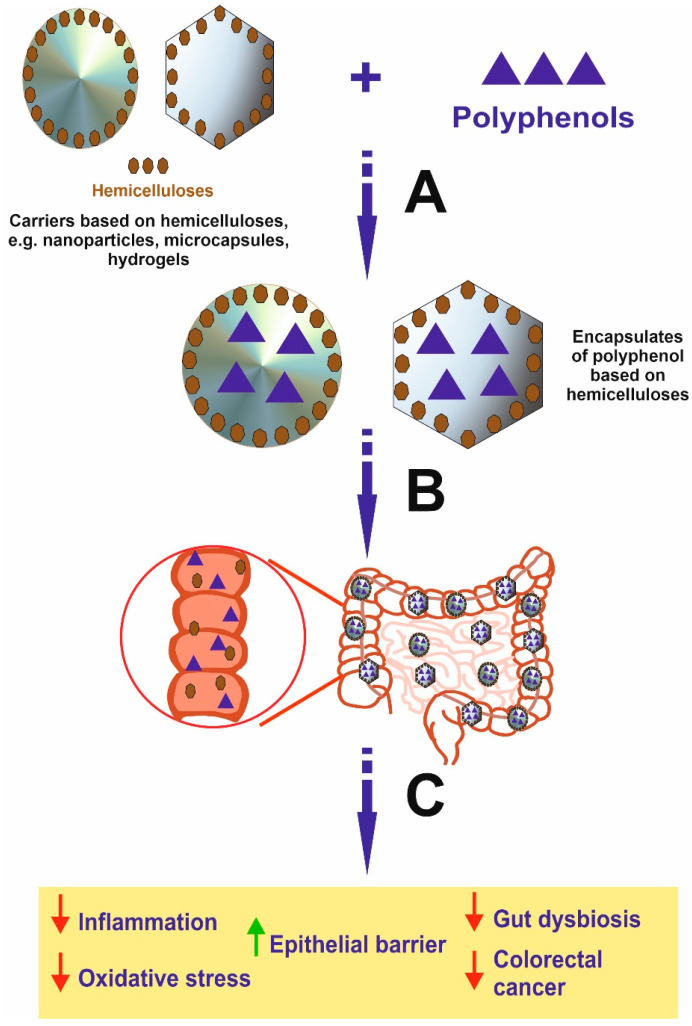
Schematic view presenting the formation of polyphenol encapsulates based on hemicelluloses and effects induced by these compounds in the colon. It is important to emphasize that encapsulates may be decomposed in the intestine by microbiota, leading to the release of free polyphenol and hemicellulose in the organ, where disease occurs. In addition, the effect is stronger compared to polyphenol alone and results from the activity of both polyphenol and hemicellulose, which may synergistically act. A—Encapsulation process; B—Oral intake of encapsulates of polyphenols based on hemicelluloses and the transport to the colon; C—The disintegration of encapsulates and release of free polyphenols and hemicelluloses exerting anti-cancer activity and effect for inhibition of IBD.

Encapsulation of polyphenols based on hemicelluloses, e.g., microcapsules, nanoparticles, nanoemulsions, and hydrogels, serves as a good colon-targeted delivery system for polyphenols and enhance their bioaccessibility, favoring the use of hemicellulose-based encapsulation with polyphenols as a treatment for IBD and CRC (Figure 3). Nevertheless, there is a lack of clinical studies confirming these properties; most of those described in our paper were confined to the preclinical settings and assess the properties of the encapsulates and the release of the polyphenol payload. Hence, although our findings regarding the use of encapsulation of polyphenols based on hemicelluloses are encouraging, further extensive studies using IBD and CRC models are required; these should address inter alia an assessment of their distribution and absorption into tissues, as well as a determination of the most effective dosages. Additionally, further clinical trials are needed using encapsulation of polyphenolic compounds based on hemicelluloses in IBD and CRC to confirm their effectiveness.

## Figures and Tables

**Table 1:** Overview of studies related to individual encapsulated polyphenolic compounds.

Polyphenol	Study Model	Dose/Duration	Biological Effects/Findings	Ref.
IBD				
Curcumin	DSS-induced BALB/c mice	Normal rodent diet containing 0.2% curcumin nanoparticles for 7 days before DSS administration	↓ IL-1β, IL-6, TNF-α, CXCL1, CXCL2 mRNA ↓ neutrophil infiltration ↓ histopathological score ↓ disease activity index ↓ colon weight/length ratio ↑ body weight	[69]
Curcumin	Acetic acid-induced Wistar rats	100 mg/kg curcumin nanoparticles orally through a gastric tube daily for 2 weeks	↑ goblet cells ↑ crypts ↓ inflammatory cells infiltration ↓ IL-6, TNF-α, TGF-β mRNA	[70]
Curcumin	DSS-induced FVB male mice	nanoparticles containing curcumin and catalase with poly(lactic-co-glycolic acid)-based surface functionalized with pluronic F127—5 mg/kg daily as equivalent of curcumin for 7 days	↓ IL-6, IL-12, TNF-α protein ↑ IL-10 protein ↓ MPO activity ↑ colon length ↑ body weight	[71]
Curcuminoids	randomized double-blind controlled trial; Fifty-six patients with the diagnosis of mild to moderate UC; treatment group (n = 28) or placebo group (n = 28)	curcuminoids nanomicelles (80 mg, three times daily, orally) plus mesalamine (3 g/24 h, orally)—the treatment group placebo plus mesalamine—the control group for 4 weeks	↓ symptoms ↓ disease activity	[72]
EGCG	DSS-induced C57BL/6J mice	EGCG hydrogels daily by oral gavage for 7 days (150 mg/kg/day—dose of EGCG)	↓ serum IL-1β, IL-6, TNF-α, IFN-γ protein ↓ colonic IL-1β, IL-6, TNF-α, IFN-γ mRNA ↑ ZO-1, Claudin-1, Occludin protein ↑ cecal SCFAs level ↓ disease activity index ↑ colon length ↑ body weight	[73]
Oleuropein	DSS-induced C57BL/6 mice	1.7 g of oleuropein loaded into nanostructured lipid carries by oral gavage for 5 days	↓ IL-6, TNF-α protein ↓ MPO activity ↓ ROS level	[66]
Resveratrol	Acetic acid-induced Wistar rats	25 mg/kg resveratrol-loaded microsponges orally for 7 days	↓ microscopic colon damage	[59]
	TNBS-induced Wistar rats	1 mg in 8 mg nanoparticles intrarectally for 7 days	↓ IL-1β, IL-6, IL-12, TNF-α, MCP-1, ICAM-1 mRNA ↑ Muc-2, Muc-3, villin mRNA ↓ MPO activity ↑ GSH content ↓ colon weight/length ratio	[60]
	Winnie mice	Resveratrol-loaded nanoparticles daily by oral gavage for 14 days	↓ IL-17 mRNA ↑ IL-10 mRNA ↓ histopathological score ↓ disease activity index ↑ body weight	[61]
	DSS-induced C57BL/6J mice	40, 80 mg/kg nanoparticles with resveratrol and poly (D,L-lactide-co-glycolide) deposited with chitosan and alginate daily for 3 days	↑ colon length ↓ disease activity index ↑ body weight	[63]
	Oxazolone-induced Wistar rats	10 mg/kg by oral gavage for 14 days	↓ MPO activity ↓ caspase-3 activity ↓ disease activity index ↓ histopathological score	[64]
Rosmarinic acid	DSS-induced C57BL/6 mice	Intravenous (retro-orbital) injection of 10, 20, 30 mg/kg nanoparticles with rosmarinic acid every other day for 10 days	↓ IL-1β, IL-6, IL-12, TNF-α, IFN-γ protein ↓ MPO activity ↓ histopathological score ↑ colon length ↓ disease activity index ↑ body weight	[65]
	DSS-induced C57BL/6 mice	5, 10, 20 mg/kg rosmarinic acid-loaded nanovesicles orally as pretreatment from three days prior to colitis induction and during days 1, 3, 5, and 7 of DSS administration	↓ TNF-α protein ↓ MPO activity ↓ NLRP3, cas-1, ASC protein ↑ Nrf2, HO-1 protein ↓ histopathological score ↓ disease activity index ↓ colon weight/length ratio ↑ body weight	[67]
Silymarin	DSS-induced ICR mice	30 mg/kg silica-installed redox nanoparticles with silymarin daily orally for 7 days	↓ histopathological score ↓ disease activity index ↑ colon length ↑ body weight	[68]
CRC				
Curcumin	Caco-2 cells	20, 25, 30, 35 µM curcumin-poly (allyl amine) hydrochloride based nanocapsules for 24, 48 h	↓ cell viability	[76]
	DMH-stimulated Balb/c mice	curcumin-poly (allyl amine) hydrochloride based nanocapsules (1:2 curcumin: poly (allyl amine) hydrochloride) for 6 weeks (5 days/week)	↓ COX-2 protein ↓ iNOS activity	
Curcumin	HCT-116, SW-620 cells	0–25 µg/mL curcumin-encapsulated fusion protein-based nanocarrier for 24 h	↓ cell viability	[77]
Ferulic acid andResveratrol	HT-29 cells	0–30 µg/mL trans-resveratrol-ferulic acid loaded chitosan coated folic acid conjugate solid lipid nanoparticles (equivalent to 2 mg resveratrol and ferulic acid) for 24, 48 h	↑ apoptosis ↑ Bax, cytochrome-C, p53 protein ↓ Cyclin B, Cyclin D1, Cyclin E, Cdk-2, -4, -6 protein	[83]
Resveratrol	HT-29 cells	50, 100, 200 µM resveratrol loaded liposomes for 24 h	↓ cell viability	[79]
Resveratrol	HT-29 cells	resveratrol loaded nanocapsules for 24, 48 h	↑ apoptosis	[80]
Resveratrol	HT-29, LS147T, LPS-stimulated RAW264.7 cells	encapsulated resveratrol in colloidal mesoporous silica nanoparticles (resveratrol at concentrations of 100, 200, 400 µM) for 6, 48 h	↓ cell viability ↓ NF-κB	[78]
Resveratrol	SW-480, CT-26 cells	5–160 µg/mL oxaliplatin- and resveratrol-loaded N,O-carboxymethyl chitosan nanoparticles for 24, 48 h	↓ cell viability	[82]
	BALB/c mice with subcutaneously injected CT-26 cells	oxaliplatin- and resveratrol-loaded N,O-carboxymethyl chitosan nanoparticles (equivalent of 8 and 30 mg/kg of oxaliplatin and resveratrol, respectively) once every 2 days for a total of 10 treatment via a tail vein injection	↓ tumor weight ↓ tumor volume ↑ apoptosis ↓ α-SMA, CUGBP1 protein	
Resveratrol	HCT-116, HT-29, Caco-2 cells	Zein resveratrol nanoparticles for 2, 4, 24, 48 h	↓ cell viability ↑ ROS activity ↑ eNOS level ↑ apoptosis ↓ NF-κB mRNA ↑ caspase-3, cleaved caspase-3 mRNA	[81]
Tannic acid	LPS-stimulated RAW264.7 cells; HT-29 cells	tannic acid containing nanoparticles at an equal tannic acid concentration of 10 µg/mL for 0.5, 1, 2, 24 h for cells	↓ ROS level ↓ IL-6, TNF-α protein	[75]
	AOM/DSS-induced C57/BL6 mice	tannic acid containing nanoparticles at an equal tannic acid concentration of 25 mg/kg by oral gavage once a day for two seven day-cycles	↓ histopathological score ↓ disease activity index ↓ tumor size ↑ colon length ↑ body weight	

Legend: ↑ activation or increase; ↓ inhibition or decrease.

**Table 2:** Overview of studies related to hemicellulose-based carriers and encapsulates of polyphenols based on hemicelluloses.

Hemicellulose	Encapsulate/Carrier Type	Characteristic Encapsulation/Carrier	Testing Method/Study Model	Biological Effects	Ref.
CARRIERS					
Arabinoxylan from dried distillers’ grains with solubles	Gels prepared by coaxial electrospraying	Mean diameter 533 ± 136 μm; rheological parameters: storage (G’) moduli 293 Pa and loss moduli (G”) 0.31 Pa	In vitro characterization; CCD 841 CoN cells	↔ cell proliferation	[167]
Xylan	Porphyrin-xylan-coated silica nanoparticles	Spherical shape nanoparticles with an average diameter of 80 nm; the hydrodynamic diameter of 78.43 ± 19.92 nm with a 0.062 polydispersity index; zeta potential—the presence of negative charges on the surface	In vitro characterization; HCT-116, HT-29 cells	↓ cell viability	[168]
Xylan	Porphyrin-xylan-coated silica nanoparticles	as above	HT-29 tumor-bearing Balb/c nude mice	↓ tumour volume	[169]
Baggase xylan	Xylan/andrographolide folate-g-dimethylaminoethyl methacrylate/diethylene glycol dimethacrylate nanoparticles prepared by the nanoprecipitation method	Spherical morphology nanoparticles with size of 100–200 nm; the estimated free energy of binding ranges from −0.62 kcal/mol to −4.12 kcal/mol; final intermolecular energy −14.56 to −11.06 kcal/mol	In vitro characterization; BEL-7407, NCI-H460, MGC80-3B, BEAS-2B cells	↓ cell viability	[170]
Baggase xylan	Xylan/andrographolide grafted and esterified derivative nanoparticles	Spherical morphology nanoparticles with size of about 100 nm; the degree of esterification substitution of 0.43; the grafting rate of the product of 42%; the estimated free energy of binding ranges from −8.94 kcal/mol to −14.68 kcal/mol; final intermolecular energy −18.85 to −13.11 kcal/mol	In vitro characterization; BEL-7407, MDA-MB-231, MGC80-3B, LO_2_ cells	↓ cell viability	[171]
Xylan from corn cobs	Xylan-based microparticles prepared by crosslinking polymerization using sodium trimetaphosphate	Narrow monodisperse size distributions with mean sizes being between 3.5 and 12.5 μm in dried state	In vitro characterization	NA	[172]
ENCAPSULATES					
Water-extractable arabinoxylans from rye bran	Honey polyphenol-loaded microcapsules prepared by spray drying	Spherical and homogeneous surfaces; the water activity of the microcapsules ranged from 0.115 to 0.218; the obtained microcapsules had average inner cell dimensions of approximately 1.92–11.16 μm	In vitro characterization	NA	[159]
Water-extractable arabinoxylans from rye bran	Honey polyphenol-loaded microcapsules prepared by spray drying	As above; the ratio of core material to the carrier was 1:4	LPS-stimulated RAW264.7 cells; NIH-3T3 cells	↓ IL-6, TNF-α protein ↓ NO level ↓ cell migration	[160]
Xylan from corn cobs	Xylan-curcumin conjugate nanoparticles	The average particle size 253 nm; the zeta potential of −18.76 mV; the yield of nanoparticles was 87%	In vitro characterization; HT-29, HCT-115 cells	↓ cell viability	[162]
Xylan from agro waste corn-cob	Xylan-curcumin conjugate nanoparticles synthesized via covalent conjugation of curcumin to xylan through a disulphide (-S-S-) linkage with (I) or without (II) assembled lipophilic 5-fluorouracil-stearic acid through dialysis membrane method	The appropriate size (~217 ± 2.52 nm); high drug loading of curcumin (~31.4 wt%); zeta potential value −17.33 ± 0.88 mV (for II) and −17.12 ± 1.12 mV (for I)	In vitro characterization; HT-29, HCT-115 cells	↓ cell viability	[163]
Bagasse xylan	Bagasse xylan/resveratrol graft-esterified composite nanoparticles	Spherical structure with an average particle size of about 100 nm; the estimated free energy of binding ranges from −3.24 kcal/mol to −6.3 kcal/mol;	In vitro characterization; BEL-7407, NGEC, MGC80-3, NCI-H460 cells	↓ cell viability	[164]
Xylan from corn	Chemically crosslinked xylan–β-Cyclodextrin hydrogel loaded into curcumin and 5-FU synthesized using ethylene glycol diglycidyl ether as a crosslinker in alkaline medium at different molar ratio	The loading of 98% of 5-FU and 26% of curcumin; the cumulative release of 56% 5-FU and 37% curcumin after 24 h	In vitro characterization	NA	[165]
Wheat bran arabinoxylan	Microhydrogels with gallic acid prepared by enzymatic and coacervation	The particle size ranges of 469–678 nm; enzymatically produced hydrogels attained higher zeta potential (−8.8 mV) and released gallic acid with anti-oxidant capacity of 91%	In vitro characterization	NA	[166]
Konjac glucomannan	Konjac glucomannan octenyl succinate nanoemulsions loaded into curcumin	Spherical structure with a rough matte edge morphology; the size was 94.2 ± 4.1 nm; The polydispersity index was 0.258 ± 0.010; loading capacity (1.25 ± 0.03 mg/mL); the zeta potential −11.5 ± 1.7 mV;	In vitro and in vivo characterization	NA	[174]
Konjac glucomannan	Multilayered emulsions with coating carboxymethyl konjac glucomannan and loaded curcumin	The size ranges about from 180 to 1100 nm; the zeta potential ranges about from −27 to −12 mV; the encapsulation efficiency was about 90%	In vitro and in vivo characterization	NA	[175]
Konjac glucomannan	Carboxymethyl konjac glucomannan/chitosan complex nanogels with loaded curcumin	Uncrosslinked nanogels: size- 259.2–987.26 nm, the polydispersity index: 0.24–0.36; swelling capacity: 31.51–77.67 (depending on pH); crosslinked nanogels: size- 233.54–883.47 nm; the polydispersity index: 0.25–0.31; swelling capacity: 25.24–68.14 (depending on pH);	In vitro characterization	NA	[176]
Konjac glucomannan	Microcapsules with anthocyanins from hibiscus prepared by spray drying and freeze drying techniques	The encapsulation efficiency 43.6%	In vitro characterization	NA	[177]
Guar gum	Double emulsions W/O/W with anthocyanins	The encapsulation efficiency 90.6%; thermal degradation constant—0.04–0.0805 (depending on the concentration of guar gum); the spectral characteristics of dispersions—λmax 534–568 (depending on the concentration of guar gum)	In vitro characterization	NA	[178]
Guar gum	Microcapsules prepared using partially hydrolyzed guar gum by spray-drying and freeze-drying with grape (*Vitis labrusca* var. Bordo) skin phenolic extract	Average diameter 8.48–684.85 μm, particle size distribution (Span) 1.94–5.99, total phenolics 21.37–23.39 mg GAE/g dry basis, total anthocyanins 17.07–21.05 mg malvidin-3,5-diglucoside/g dry basis, DPPH capacity 50.82–73.42, HRSA 82.2–84.4% (depending on the concentration of guar gum and encapsulation method);	In vitro characterization	NA	[179]
Guar gum	Microcapsules containing anthocyanins from chokeberry with guar gum as wall material	Encapsulation efficiency 92.98 ± 0.87%; water solubility 90.7 ± 0.1; moisture content 1.66 ± 0.002%; particle size 16.29 ± 0.02 μm; % of degradation during 7 storage—5.81%	In vitro characterization	NA	[180]
Oat β-glucan	Curcumin loaded in octenylsuccinate oat β-glucan micelles	Elliptical in shape; the maximum curcumin loading capacity value of the micelle was obtained as 4.21 μg/mg; the average size 308 nm; the zeta potential −10.8 mV	In vitro characterization	NA	[182]
Oat β-glucan	Self-aggregates of octenylsuccinate oat β-glucan-based nanocapsules with loaded into curcumin	The size—214.3–509.6 nm; the polydispersity index—0.185–0.477; the curcumin retention—38.2–100.0 (depending on heating time, temperature, light, freeze–thaw cycle)	In vitro characterization	NA	[183]
Yeast β-1,3-glucan	Particles with loaded into curcumin	Various shapes; however, often they have asymmetric rod-like shape; the mean size (diameter)—5.8 ± 3 μm	In vitro characterization; LPS-stimulated THP-1-XBlue™- MD2-CD14 cells; THP-1 cells	↓ IL-1β, TNF-α protein ↓ NF-κB/AP-1 activity	[184]
Yeast β-1,3/1,6-glucans	Glucan particles with incorporated curcumin; incorporation was performed by the slurry evaporation method	The real mass fraction of curcumin in the composites was found to be 20.47% ± 0.65%	In vitro characterization; DSS-induced Wistar rats	↓ IL-1β, IL-6, TNF-α protein ↔ IL-10, IL-17, SOD-2 protein ↔ MMP-9 production ↔ MPO activity	[185]
Glucan from *Agaricus bisporous*	Curcumin loaded into chitin–glucan quercetin conjugate	Flaky nature after grafting quercetin surface become change; the entrapment efficiency 77.32%; DPPH 74.26 mg/mL, ABTS 82.86 mg/mL	In vitro characterization; J774 cells	↓ cell viability	[186]
Yeast glucan	Glucan particles with incorporated trans-resveratrol/EGCG by slurry rotary evaporation and spray drying	EGCG—composites (*w*/*w*): 51.5% (rotary evaporation), 61.8% (spray drying); resveratrol—composites (*w*/*w*): 114.7% (rotary evaporation), 138.2% (spray drying);	In vitro characterization; LPS-stimulated THP-1-XBlue™- MD2-CD14 cells	↓ TNF-α protein ↓ NF-κB/AP-1 activity	[187]
Yeast β-1,3-D-glucan	Glucan microcapsules with loaded into EGCG and berberine	The elliptical structure with pores; the particle size 3117.8 ± 220.6 nm; the encapsulation efficiency for EGCG 92.74 ± 0.1%	In vitro characterization; DSS-induced C57BL/6 mice	↓ IL-1β, TNF-α protein ↓ H_2_O_2_ concentration ↓ MDA level ↓ histological score ↑ body weight ↑ colon length	[188]
β-glucan from barley	Glucan microcapsules with loaded into saffron anthocyanins by spray drying	The powder yield 45.33%; the encapsulation efficiency 45.00 ± 1.2%; bulk density 0.419 ± 0.11; tapped density 0.543 ± 0.31; the 90% of microparticles had size less than 391.31 μm	In vitro characterization	NA	[189]

Legend: ↑ activation or increase; ↓ inhibition or decrease; ↔ no impact; NA—not applicable.

## Data Availability

Not applicable.

## Abbreviations

5-ASA	5-aminosalicylic acid
AOM	azoxymethane
CD	Crohn’s disease
COX-2	cyclooxygenase 2
CRC	colorectal cancer
DSS	dextran sulphate sodium
EGCG	epigallocatechin gallate
IBD	inflammatory bowel diseases
IL	interleukin
iNOS	inducible nitric oxide synthase
NF-κB	nuclear factor kappa B
NO	nitric oxide
Nrf2	nuclear factor erythroid 2-related factor 2
ROS	reactive oxygen species
SCFA	short chain fatty acids
TNBS	2,4,6-trinitrobenzene sulfonic acid
UC	ulcerative colitis

## References

[1] Mak W.Y., Zhao M., Ng S.C., Burisch J. (2020). The Epidemiology of Inflammatory Bowel Disease: East Meets West. J. Gastroenterol. Hepatol..

[2] Agrawal M., Spencer E.A., Colombel J.F., Ungaro R.C. (2021). Approach to the Management of Recently Diagnosed Inflammatory Bowel Disease Patients: A User’s Guide for Adult and Pediatric Gastroenterologists. Gastroenterology.

[3] Kaplan G.G., Windsor J.W. (2021). The four epidemiological stages in the global evolution of inflammatory bowel disease. Nat. Rev. Gastroenterol. Hepatol..

[4] Seyedian S.S., Nokhostin F., Malamir M.D. (2019). A Review of the Diagnosis, Prevention, and Treatment Methods of Inflammatory Bowel Disease. J. Med. Life.

[5] Eaden J.A., Abrams K.R., Mayberry J.F., Eaden J.A., Abrams K.R., Mayberry J.F. (2001). The Risk of Colorectal Cancer in Ulcerative Colitis: A Meta-Analysis. Gut.

[6] Nadeem M.S., Kumar V., Al-Abbasi F.A., Kamal M.A., Anwar F. (2020). Risk of Colorectal Cancer in Inflammatory Bowel Diseases. Semin. Cancer Biol..

[7] Shah S.C., Itzkowitz S.H. (2022). Colorectal Cancer in Inflammatory Bowel Disease: Mechanisms and Management. Gastroenterology.

[8] Dekker E., Tanis P.J., Vleugels J.L.A., Kasi P.M., Wallace M.B. (2019). Colorectal Cancer. Lancet.

[9] Baidoun F., Elshiwy K., Elkeraie Y., Merjaneh Z., Khoudari G., Sarmini M.T., Gad M., Al-Husseini M., Saad A. (2020). Colorectal Cancer Epidemiology: Recent Trends and Impact on Outcomes. Curr. Drug Targets.

[10] Morgan E., Arnold M., Gini A., Lorenzoni V., Cabasag C.J., Laversanne M., Vignat J., Ferlay J., Murphy N., Bray F. (2023). Global burden of colorectal cancer in 2020 and 2040: Incidence and mortality estimates from GLOBOCAN. Gut.

[11] Brumatti L.V., Marcuzzi A., Tricarico P.M., Zanin V., Girardelli M., Bianco A.M. (2014). Curcumin and Inflammatory Bowel Disease: Potential Andlimits of Innovative Treatments. Molecules.

[12] Park K.T., Ehrlich O.G., Allen J.I., Meadows P., Szigethy E.M., Henrichsen K., Kim S.C., Lawton R.C., Murphy S.M., Regueiro M. (2020). The Cost of Inflammatory Bowel Disease: An Initiative from the Crohn’s & Colitis Foundation. Inflamm. Bowel Dis..

[13] Roberti R., Iannone L.F., Palleria C., De Sarro C., Spagnuolo R., Barbieri M.A., Vero A., Manti A., Pisana V., Fries W. (2020). Safety Profiles of Biologic Agents for Inflammatory Bowel Diseases: A Prospective Pharmacovigilance Study in Southern Italy. Curr. Med. Res. Opin..

[14] Poturnajova M., Furielova T., Balintova S., Schmidtova S., Kucerova L., Matuskova M. (2021). Molecular Features and Gene Expression Signature of Metastatic Colorectal Cancer (Review). Oncol. Rep..

[15] Fraga C.G., Croft K.D., Kennedy D.O., Tomás-Barberán F.A. (2019). The Effects of Polyphenols and Other Bioactives on Human Health. Food Funct..

[16] Chojnacka K., Lewandowska U. (2018). Chemopreventive Effects of Polyphenol-Rich Extracts against Cancer Invasiveness and Metastasis by Inhibition of Type IV Collagenases Expression and Activity. J. Funct. Foods.

[17] Caban M., Lewandowska U. (2022). Polyphenols and the Potential Mechanisms of Their Therapeutic Benefits against Inflammatory Bowel Diseases. J. Funct. Foods.

[18] Chojnacka K., Owczarek K., Caban M., Sosnowska D., Kajszczak D., Lewandowska U. (2022). Chemopreventive Effects of Japanese Quince (*Chaenomeles japonica* L.) Phenol Leaf Extract on Colon Cancer Cells Through the Modulation of Extracellular Signal-Regulated Kinases/Akt Signaling Pathway. J. Physiol. Pharmacol..

[19] Owczarek K., Sosnowska D., Kajszczak D., Lewandowska U. (2022). Evaluation of Phenolic Composition, Antioxidant and Cytotoxic Activity of Aronia Melanocarpa Leaf Extracts. J. Physiol. Pharmacol..

[20] Caban M., Owczarek K., Chojnacka K., Podsedek A., Sosnowska D., Lewandowska U. (2022). Chemopreventive Properties of Spent Hops (*Humulus lupulus* L.) Extract Against Angiogenesis, Invasion and Migration of Colorectal Cancer Cells. J. Physiol. Pharmacol..

[21] Zhang Y., Peng L., Li W., Dai T., Nie L., Xie J., Ai Y., Li L., Tian Y., Sheng J. (2020). Polyphenol Extract of Moringa Oleifera Leaves Alleviates Colonic Inflammation in Dextran Sulfate Sodium-Treated Mice. Evid.-Based Complement. Altern. Med..

[22] Marzo F., Milagro F.I., Barrenetxe J., Díaz M.T., Martínez J.A. (2021). Azoxymethane-Induced Colorectal Cancer Mice Treated with a Polyphenol-Rich Apple Extract Show Less Neoplastic Lesions and Signs of Cachexia. Foods.

[23] Wu Z., Huang S., Li T., Li N., Han D., Zhang B., Xu Z.Z., Zhang S., Pang J., Wang S. (2021). Gut Microbiota from Green Tea Polyphenol-Dosed Mice Improves Intestinal Epithelial Homeostasis and Ameliorates Experimental Colitis. Microbiome.

[24] Guo X., Xu Y., Geng R., Qiu J., He X. (2022). Curcumin Alleviates Dextran Sulfate Sodium-Induced Colitis in Mice Through Regulating Gut Microbiota. Mol. Nutr. Food Res..

[25] Lewandowska U., Szewczyk K., Hrabec E., Janecka A., Gorlach S. (2013). Overview of Metabolism and Bioavailability Enhancement of Polyphenols. J. Agric. Food Chem..

[26] Yvonne K., Heikki V., Johanna T., Bjarne H., Jenni B. (2017). Galactoglucomannan-Rich Hemicellulose Extract from Norway Spruce (*Picea abies*) Exerts Benefeffects on Chronic Prostatic Inflammation and Lower Urinary Tract Symptoms in Vivo. Int. J. Biol. Macromol..

[27] Badr El-Din N.K., Ali D.A., Othman R., French S.W., Ghoneum M. (2020). Chemopreventive Role of Arabinoxylan Rice Bran, MGN-3/Biobran, on Liver Carcinogenesis in Rats. Biomed. Pharmacother..

[28] Luo S., He L., Zhang H., Li Z., Liu C., Chen T. (2022). Arabinoxylan from Rice Bran Protects Mice against High-Fat Diet-Induced Obesity and Metabolic Inflammation by Modulating Gut Microbiota and Short-Chain Fatty Acids. Food Funct..

[29] Qiu A., Wang Y., Zhang G., Wang H. (2022). Natural Polysaccharide-Based Nanodrug Delivery Systems for Treatment of Diabetes. Polymers.

[30] Brglez Mojzer E., Knez Hrnčič M., Škerget M., Knez Ž., Bren U. (2016). Polyphenols: Extraction Methods, Antioxidative Action, Bioavailability and Anticarcinogenic Effects. Molecules.

[31] Zeeshan M., Ali H., Khan S., Khan S.A., Weigmann B. (2019). Advances in Orally-Delivered PH-Sensitive Nanocarrier Systems; an Optimistic Approach for the Treatment of Inflammatory Bowel Disease. Int. J. Pharm..

[32] Bassotti G., Antonelli E., Villanacci V., Nascimbeni R., Dore M.P., Pes G.M., Maconi G. (2020). Abnormal Gut Motility in Inflammatory Bowel Disease: An Update. Tech. Coloproctol..

[33] Tie S., Tan M. (2022). Current Advances in Multifunctional Nanocarriers Based on Marine Polysaccharides for Colon Delivery of Food Polyphenols. J. Agric. Food Chem..

[34] Lin J.C., Wu J.Q., Wang F., Tang F.Y., Sun J., Xu B., Jiang M., Chu Y., Chen D., Li X. (2019). QingBai Decoction Regulates Intestinal Permeability of Dextran Sulphate Sodium-Induced Colitis through the Modulation of Notch and NF-ΚB Signalling. Cell Prolif..

[35] Li X., Lu C., Yang Y., Yu C., Rao Y. (2020). Site-Specific Targeted Drug Delivery Systems for the Treatment of Inflammatory Bowel Disease. Biomed. Pharmacother..

[36] Zhu L., Shen H., Gu P., Liu Y., Zhang L., Cheng J. (2020). Baicalin Alleviates TNBS-induced Colitis by Inhibiting PI3K/AKT Pathway Activation. Exp. Ther. Med..

[37] Tena N., Martín J., Asuero A.G. (2020). State of the Art of Anthocyanins: Antioxidant Activity, Sources, Bioavailability, and Therapeutic Effect in Human Health. Antioxidants.

[38] Flynn S., Eisenstein S. (2019). Inflammatory Bowel Disease Presentation and Diagnosis. Surg. Clin. N. Am..

[39] Gan R.Y., Li H.B., Sui Z.Q., Corke H. (2018). Absorption, Metabolism, Anti-Cancer Effect and Molecular Targets of Epigallocatechin Gallate (EGCG): An Updated Review. Crit. Rev. Food Sci. Nutr..

[40] Guan Q. (2019). A Comprehensive Review and Update on the Pathogenesis of Inflammatory Bowel Disease. J. Immunol. Res..

[41] Abdelhalim K.A., Uzel A., Gülşen Ünal N. (2020). Virulence Determinants and Genetic Diversity of Adherent-Invasive Escherichia Coli (AIEC) Strains Isolated from Patients with Crohn’s Disease. Microb. Pathog..

[42] Xu N., Bai X., Cao X., Yue W., Jiang W., Yu Z. (2021). Changes in Intestinal Microbiota and Correlation with TLRs in Ulcerative Colitis in the Coastal Area of Northern China. Microb. Pathog..

[43] Miao F. (2022). Hydroxytyrosol Alleviates Dextran Sodium Sulfate–Induced Colitis by Inhibiting NLRP3 Inflammasome Activation and Modulating Gut Microbiota in Vivo. Nutrition.

[44] Wong S.H., Yu J. (2019). Gut Microbiota in Colorectal Cancer: Mechanisms of Action and Clinical Applications. Nat. Rev. Gastroenterol. Hepatol..

[45] Alrafas H.R., Busbee P.B., Nagarkatti M., Nagarkatti P.S. (2019). Resveratrol Modulates the Gut Microbiota to Prevent Murine Colitis Development through Induction of Tregs and Suppression of Th17 Cells. J. Leukoc. Biol..

[46] Gómez-López I., Lobo-Rodrigo G., Portillo M.P., Cano M.P. (2021). Characterization, Stability, and Bioaccessibility of Betalain and Phenolic Compounds from Opuntia Stricta Var. Dillenii Fruits and Products of Their Industrialization. Foods.

[47] Di Lorenzo C., Colombo F., Biella S., Stockley C., Restani P. (2021). Polyphenols and Human Health: The Role of Bioavailability. Nutrients.

[48] Anselmo A.C., Gokarn Y., Mitragotri S. (2018). Non-Invasive Delivery Strategies for Biologics. Nat. Rev. Drug Discov..

[49] Ahadian S., Finbloom J.A., Mofidfar M., Diltemiz S.E., Nasrollahi F., Davoodi E., Hosseini V., Mylonaki I., Sangabathuni S., Montazerian H. (2020). Micro and Nanoscale Technologies in Oral Drug Delivery. Adv. Drug Deliv. Rev..

[50] Murakami A. (2014). Dose-Dependent Functionality and Toxicity of Green Tea Polyphenols in Experimental Rodents. Arch. Biochem. Biophys..

[51] Samba-Mondonga M., Constante M., Fragoso G., Calvé A., Santos M.M. (2019). Curcumin Induces Mild Anemia in a DSS-Induced Colitis Mouse Model Maintained on an Iron-Sufficient Diet. PLoS ONE.

[52] Lambert J.D., Kennett M.J., Sang S., Reuhl K.R., Ju J., Yang C.S. (2010). Hepatotoxicity of High Oral Dose (−)-Epigallocatechin-3-Gallate in Mice. Food Chem. Toxicol..

[53] Inoue H., Akiyama S., Maeda-Yamamoto M., Nesumi A., Tanaka T., Murakami A. (2011). High-Dose Green Tea Polyphenols Induce Nephrotoxicity in Dextran Sulfate Sodium-Induced Colitis Mice by down-Regulation of Antioxidant Enzymes and Heat-Shock Protein Expressions. Cell Stress Chaperones.

[54] Kobayashi H., Murata M., Kawanishi S., Oikawa S. (2020). Polyphenols with Anti-Amyloid β Aggregation Show Potential Risk of Toxicity via pro-Oxidant Properties. Int. J. Mol. Sci..

[55] Posadino A.M., Cossu A., Giordo R., Zinellu A., Sotgia S., Vardeu A., Hoa P.T., Van Nguyen L.H., Carru C., Pintus G. (2015). Resveratrol Alters Human Endothelial Cells Redox State and Causes Mitochondrial-Dependent Cell Death. Food Chem. Toxicol..

[56] Liu Y., Wu X., Hu X., Chen Z., Liu H., Takeda S., Qing Y. (2017). Multiple Repair Pathways Mediate Cellular Tolerance to Resveratrol-Induced DNA Damage. Toxicol. Vitr..

[57] Chai R., Chen Y., Yuan H., Wang X., Guo S., Qi J., Zhang H., Zhan Y., An H. (2017). Identification of Resveratrol, an Herbal Compound, as an Activator of the Calcium-Activated Chloride Channel, TMEM16A. J. Membr. Biol..

[58] Inoue H., Maeda-Yamamoto M., Nesumi A., Tanaka T., Murakami A. (2013). Low and Medium but Not High Doses of Green Tea Polyphenols Ameliorated Dextran Sodium Sulfate-Induced Hepatotoxicity and Nephrotoxicity. Biosci. Biotechnol. Biochem..

[59] Gandhi H., Rathore C., Dua K., Vihal S., Tambuwala M.M., Negi P. (2020). Efficacy of Resveratrol Encapsulated Microsponges Delivered by Pectin Based Matrix Tablets in Rats with Acetic Acid-Induced Ulcerative Colitis. Drug Dev. Ind. Pharm..

[60] Lozano-Pérez A.A., Rodriguez-Nogales A., Ortiz-Cullera V., Algieri F., Garrido-Mesa J., Zorrilla P., Rodriguez-Cabezas M.E., Garrido-Mesa N., Pilar Utrilla M., de Matteis L. (2014). Silk Fibroin Nanoparticles Constitute a Vector for Controlled Release of Resveratrol in an Experimental Model of Inflammatory Bowel Disease in Rats. Int. J. Nanomed..

[61] Pujara N., Wong K.Y., Qu Z., Wang R., Moniruzzaman M., Rewatkar P., Kumeria T., Ross B.P., McGuckin M., Popat A. (2021). Oral Delivery of β-Lactoglobulin-Nanosphere-Encapsulated Resveratrol Alleviates Inflammation in Winnie Mice with Spontaneous Ulcerative Colitis. Mol. Pharm..

[62] Iglesias N., Galbis E., Díaz-Blanco M.J., Lucas R., Benito E., De-Paz M.V. (2019). Nanostructured Chitosan-Based Biomaterials for Sustained and Colon-Specific Resveratrol Release. Int. J. Mol. Sci..

[63] Jin M., Li S., Wu Y., Li D., Han Y. (2021). Construction of Chitosan/Alginate Nano-Drug Delivery System for Improving Dextran Sodium Sulfate-Induced Colitis in Mice. Nanomaterials.

[64] Abdin A.A. (2013). Targeting Sphingosine Kinase 1 (SphK1) and Apoptosis by Colon-Specific Delivery Formula of Resveratrol in Treatment of Experimental Ulcerative Colitis in Rats. Eur. J. Pharmacol..

[65] Chung C.H., Jung W., Keum H., Kim T.W., Jon S. (2020). Nanoparticles Derived from the Natural Antioxidant Rosmarinic Acid Ameliorate Acute Inflammatory Bowel Disease. ACS Nano.

[66] Huguet-Casquero A., Xu Y., Gainza E., Pedraz J.L., Beloqui A. (2020). Oral Delivery of Oleuropein-Loaded Lipid Nanocarriers Alleviates Inflammation and Oxidative Stress in Acute Colitis. Int. J. Pharm..

[67] Marinho S., Illanes M., Ávila-Román J., Motilva V., Talero E. (2021). Anti-Inflammatory Effects of Rosmarinic Acid-Loaded Nanovesicles in Acute Colitis through Modulation of NLRP3 Inflammasome. Biomolecules.

[68] Nguyen T.H.T., Trinh N.T., Tran H.N., Tran H.T., Le P.Q., Ngo D.N., Tran-Van H., Van Vo T., Vong L.B., Nagasaki Y. (2021). Improving Silymarin Oral Bioavailability Using Silica-Installed Redox Nanoparticle to Suppress Inflammatory Bowel Disease. J. Control. Release.

[69] Ohno M., Nishida A., Sugitani Y., Nishino K., Inatomi O., Sugimoto M., Kawahara M., Andoh A. (2017). Nanoparticle Curcumin Ameliorates Experimental Colitis via Modulation of Gut Microbiota and Induction of Regulatory T Cells. PLoS ONE.

[70] Arafat E.A., Marzouk R.E., Mostafa S.A., Hamed W.H.E. (2021). Identification of the Molecular Basis of Nanocurcumin-Induced Telocyte Preservation within the Colon of Ulcerative Colitis Rat Model. Mediat. Inflamm..

[71] Huang Y., Canup B.S.B., Gou S., Chen N., Dai F., Xiao B., Li C. (2021). Oral Nanotherapeutics with Enhanced Mucus Penetration and ROS-Responsive Drug Release Capacities for Delivery of Curcumin to Colitis Tissues. J. Mater. Chem. B.

[72] Masoodi M., Mahdiabadi M.A., Mokhtare M., Agah S., Kashani A.H.F., Rezadoost A.M., Sabzikarian M., Talebi A., Sahebkar A. (2018). The Efficacy of Curcuminoids in Improvement of Ulcerative Colitis Symptoms and Patients’ Self-Reported Well-Being: A Randomized Double-Blind Controlled Trial. J. Cell. Biochem..

[73] Hu B., Yu S., Shi C., Gu J., Shao Y., Chen Q., Li Y., Mezzenga R. (2020). Amyloid-Polyphenol Hybrid Nanofilaments Mitigate Colitis and Regulate Gut Microbial Dysbiosis. ACS Nano.

[74] Wang X., Yan J., Wang L., Pan D., Xu Y., Wang F., Sheng J., Li X., Yang M. (2020). Oral Delivery of Anti-TNF Antibody Shielded by Natural Polyphenol-Mediated Supramolecular Assembly for Inflammatory Bowel Disease Therapy. Theranostics.

[75] Le Z., He Z., Liu H., Ke J., Liu L., Liu Z., Chen Y. (2022). Orally Administrable Polyphenol-Based Nanoparticles Achieve Anti-Inflammation and Antitumor Treatment of Colon Diseases. Biomater. Sci..

[76] Slika L., Moubarak A., Borjac J., Baydoun E., Patra D. (2020). Preparation of Curcumin-Poly (Allyl Amine) Hydrochloride Based Nanocapsules: Piperine in Nanocapsules Accelerates Encapsulation and Release of Curcumin and Effectiveness against Colon Cancer Cells. Mater. Sci. Eng. C.

[77] Han Z., Song B., Yang J., Wang B., Ma Z., Yu L., Li Y., Xu H., Qiao M. (2022). Curcumin-Encapsulated Fusion Protein-Based Nanocarrier Demonstrated Highly Efficient Epidermal Growth Factor Receptor-Targeted Treatment of Colorectal Cancer. J. Agric. Food Chem..

[78] Summerlin N., Qu Z., Pujara N., Sheng Y., Jambhrunkar S., McGuckin M., Popat A. (2016). Colloidal Mesoporous Silica Nanoparticles Enhance the Biological Activity of Resveratrol. Colloids Surf. B Biointerfaces.

[79] Soo E., Thakur S., Qu Z., Jambhrunkar S., Parekh H.S., Popat A. (2016). Enhancing Delivery and Cytotoxicity of Resveratrol through a Dual Nanoencapsulation Approach. J. Colloid Interface Sci..

[80] Feng M., Zhong L.X., Zhan Z.Y., Huang Z.H., Xiong J.P. (2017). Enhanced Antitumor Efficacy of Resveratrol-Loaded Nanocapsules in Colon Cancer Cells: Physicochemical and Biological Characterization. Eur. Rev. Med. Pharmacol. Sci..

[81] Khayat M.T., Zarka M.A., El-Telbany D.F.A., El-Halawany A.M., Kutbi H.I., Elkhatib W.F., Noreddin A.M., Khayyat A.N., El-Telbany R.F.A., Hammad S.F. (2022). Intensification of Resveratrol Cytotoxicity, pro-Apoptosis, Oxidant Potentials in Human Colorectal Carcinoma HCT-116 Cells Using Zein Nanoparticles. Sci. Rep..

[82] Wang Y., Ma J., Qiu T., Tang M., Zhang X., Dong W. (2021). In Vitro and in Vivo Combinatorial Anticancer Effects of Oxaliplatin- and Resveratrol-Loaded N,O-Carboxymethyl Chitosan Nanoparticles against Colorectal Cancer. Eur. J. Pharm. Sci..

[83] Senthil Kumar C., Thangam R., Mary S.A., Kannan P.R., Arun G., Madhan B. (2020). Targeted Delivery and Apoptosis Induction of Trans-Resveratrol-Ferulic Acid Loaded Chitosan Coated Folic Acid Conjugate Solid Lipid Nanoparticles in Colon Cancer Cells. Carbohydr. Polym..

[84] Scheller H.V., Ulvskov P. (2010). Hemicelluloses. Annu. Rev. Plant Biol..

[85] Madrid Liwanag A.J., Ebert B., Verhertbruggen Y., Rennie E.A., Rautengarten C., Oikawa A., Andersen M.C.F., Clausen M.H., Scheller H.V. (2013). Pectin Biosynthesis: GALS1 in Arabidopsis Thaliana Is a β-1,4-Galactan β-1,4-Galactosyltransferase. Plant Cell.

[86] Pauly M., Gille S., Liu L., Mansoori N., de Souza A., Schultink A., Xiong G. (2013). Hemicellulose Biosynthesis. Planta.

[87] Wyman C.E., Decker S.R., Himmel M.E., Brady J.W., Skopec C., Viikari L., Dumitriu S. (2005). Hydrolysis of cellulose and hemicellulose. Polysaccharides: Structural Diversity and Functional Versatility.

[88] Cartaxo da Costa Urtiga S., Rodrigues Marcelino H., Sócrates Tabosa do Egito E., Eleamen Oliveira E. (2020). Xylan in Drug Delivery: A Review of Its Engineered Structures and Biomedical Applications. Eur. J. Pharm. Biopharm..

[89] Kishani S., Escalante A., Toriz G., Vilaplana F., Gatenholm P., Hansson P., Wagberg L. (2019). Experimental and Theoretical Evaluation of the Solubility/Insolubility of Spruce Xylan (Arabino Glucuronoxylan). Biomacromolecules.

[90] Palasingh C., Nakayama K., Abik F., Mikkonen K.S., Evenäs L., Ström A., Nypelö T. (2022). Modification of Xylan via an Oxidation–Reduction Reaction. Carbohydr. Polym..

[91] Kostalova Z., Hromádková Z., Paulsen Berit S., Ebringerová A. (2014). Bioactive Hemicelluloses Alkali-Extracted from Fallopia Sachalinensis Leaves. Carbohydr. Res..

[92] Arumugam N., Biely P., Puchart V., Gerrano A.S., De Mukherjee K., Singh S., Pillai S. (2019). Xylan from Bambara and Cowpea Biomass and Their Structural Elucidation. Int. J. Biol. Macromol..

[93] Chaves P.F.P., de Almeida S.H.P., Dallazen J.L., de Paula Werner M.F., Iacomini M., Andreatini R., Cordeiro L.M.C. (2020). Chamomile Tea: Source of a Glucuronoxylan with Antinociceptive, Sedative and Anxiolytic-like Effects. Int. J. Biol. Macromol..

[94] Voiniciuc C. (2022). Modern Mannan: A Hemicellulose’s Journey. New Phytol..

[95] Yamabhai M., Sak-Ubol S., Srila W., Haltrich D. (2016). Mannan Biotechnology: From Biofuels to Health. Crit. Rev. Biotechnol..

[96] Bulmer G.S., de Andrade P., Field R.A., van Munster J.M. (2021). Recent Advances in Enzymatic Synthesis of β-Glucan and Cellulose. Carbohydr. Res..

[97] Wu L., Zhao J., Zhang X., Liu S., Zhao C. (2021). Antitumor Effect of Soluble β-Glucan as an Immune Stimulant. Int. J. Biol. Macromol..

[98] Dutta P., Giri S., Giri T.K. (2020). Xyloglucan as Green Renewable Biopolymer Used in Drug Delivery and Tissue Engineering. Int. J. Biol. Macromol..

[99] Sarma S.M., Singh D.P., Singh P., Khare P., Mangal P., Singh S., Bijalwan V., Kaur J., Mantri S., Boparai R.K. (2018). Finger Millet Arabinoxylan Protects Mice from High-Fat Diet Induced Lipid Derangements, Inflammation, Endotoxemia and Gut Bacterial Dysbiosis. Int. J. Biol. Macromol..

[100] Zhao Z., Cheng W., Qu W., Wang K. (2020). Arabinoxylan Rice Bran (MGN-3/Biobran) Alleviates Radiation-Induced Intestinal Barrier Dysfunction of Mice in a Mitochondrion-Dependent Manner. Biomed. Pharmacother..

[101] Ghoneum M.H., El Sayed N.S. (2021). Protective Effect of Biobran/MGN-3 against Sporadic Alzheimer’s Disease Mouse Model: Possible Role of Oxidative Stress and Apoptotic Pathways. Oxid. Med. Cell. Longev..

[102] Piotrowska M., Swierczynski M., Fichna J., Piechota-Polanczyk A. (2021). The Nrf2 in the Pathophysiology of the Intestine: Molecular Mechanisms and Therapeutic Implications for Inflammatory Bowel Diseases. Pharmacol. Res..

[103] Neyrinck A.M., Possemiers S., Druart C., van de Wiele T., de Backer F., Cani P.D., Larondelle Y., Delzenne N.M. (2011). Prebiotic Effects of Wheat Arabinoxylan Related to the Increase in Bifidobacteria, Roseburia and Bacteroides/Prevotella in Diet-Induced Obese Mice. PLoS ONE.

[104] Yacoubi N., Saulnier L., Bonnin E., Devillard E., Eeckhaut V., Rhayat L., Ducatelle R., Van Immerseel F. (2018). Short-Chain Arabinoxylans Prepared from Enzymatically Treated Wheat Grain Exert Prebiotic Effects during the Broiler Starter Period. Poult. Sci..

[105] Govers C., Tang Y., Stolte E.H., Wichers H.J., Mes J.J. (2020). Wheat-Derived Arabinoxylans Reduced M2-Macrophage Functional Activity, but Enhanced Monocyte-Recruitment Capacity. Food Funct..

[106] Soufli I., Toumi R., Rafa H., Touil-Boukoffa C. (2016). Overview of Cytokines and Nitric Oxide Involvement in Immuno-Pathogenesis of Inflammatory Bowel Diseases. World J. Gastrointest. Pharmacol. Ther..

[107] Mendis M., Leclerc E., Simsek S. (2016). Arabinoxylan Hydrolyzates as Immunomodulators in Lipopolysaccharide-Induced RAW264.7 Macrophages. Food Funct..

[108] Zha Z., Lv Y., Tang H., Li T., Miao Y., Cheng J., Wang G., Tan Y., Zhu Y., Xing X. (2020). An Orally Administered Butyrate-Releasing Xylan Derivative Reduces Inflammation in Dextran Sulphate Sodium-Induced Murine Colitis. Int. J. Biol. Macromol..

[109] Owczarek K., Lewandowska U. (2017). The Impact of Dietary Polyphenols on COX-2 Expression in Colorectal Cancer. Nutr. Cancer.

[110] Badr El-Din N.K., Abdel Fattah S.M., Pan D., Tolentino L., Ghoneum M. (2016). Chemopreventive Activity of MGN-3/Biobran Against Chemical Induction of Glandular Stomach Carcinogenesis in Rats and Its Apoptotic Effect in Gastric Cancer Cells. Integr. Cancer Ther..

[111] Ooi S.L., McMullen D., Golombick T., Pak S.C. (2018). Evidence-Based Review of BioBran/MGN-3 Arabinoxylan Compound as a Complementary Therapy for Conventional Cancer Treatment. Integr. Cancer Ther..

[112] Golombick T., Diamond T.H., Manoharan A., Ramakrishna R. (2016). Addition of Rice Bran Arabinoxylan to Curcumin Therapy May Be of Benefit to Patients with Early-Stage B-Cell Lymphoid Malignancies (Monoclonal Gammopathy of Undetermined Significance, Smoldering Multiple Myeloma, or Stage 0/1 Chronic Lymphocytic Leukemia): A Preliminary Clinical Study. Integr. Cancer Ther..

[113] Mendis M., Leclerc E., Simsek S. (2017). Arabinoxylan Hydrolyzates as Immunomodulators in Caco-2 and HT-29 Colon Cancer Cell Lines. Food Funct..

[114] Paesani C., Degano A.L., Ines Z., Zalosnik M.I., Fabi J.P., Pérez G.T. (2021). Enzymatic Modification of Arabinoxylans from Soft and Hard Argentinian Wheat Inhibits the Viability of HCT-116 Cells. Food Res. Int..

[115] Li J., Jia Q., Liu Y., Chen D., Fang Z., Liu Y., Li S., Hu B., Wang C., Chen H. (2022). Different Structures of Arabinoxylan Hydrolysates Alleviated Caco-2 Cell Barrier Damage by Regulating the TLRs/MyD88/NF-ΚB Pathway. Foods.

[116] Bezerra I.d.L., Caillot A.R.C., Palhares L.C.G.F., Santana-Filho A.P., Chavante S.F., Sassaki G.L. (2018). Structural Characterization of Polysaccharides from Cabernet Franc, Cabernet Sauvignon and Sauvignon Blanc Wines: Anti-Inflammatory Activity in LPS Stimulated RAW 264.7 Cells. Carbohydr. Polym..

[117] Gu Q., Li Y., Zhen L., Zhao T., Luo L., Zhang J., Deng T., Wu M., Cheng G., Hu J. (2022). The structures of two glucomannans from Bletilla formosana and their protective effect on inflammation via inhibiting NF-κB pathway. Carbohydr. Polym..

[118] Badia R., Brufau M.T., Guerrero-Zamora A.M., Lizardo R., Dobrescu I., Martin-Venegas R., Ferrer R., Salmon H., Martínez P., Brufau J. (2012). β-Galactomannan and Saccharomyces Cerevisiae Var. Boulardii Modulate the Immune Response against Salmonella Enterica Serovar Typhimurium in Porcine Intestinal Epithelial and Dendritic Cells. Clin. Vaccine Immunol..

[119] Guo W., Gu X., Tong Y., Wang X., Wu J., Chang C. (2019). Protective Effects of Mannan/β-Glucans from Yeast Cell Wall on the Deoxyniyalenol-Induced Oxidative Stress and Autophagy in IPEC-J2 Cells. Int. J. Biol. Macromol..

[120] Zhao Y., Guo W., Gu X., Chang C., Wu J. (2020). Repression of Deoxynivalenol-Triggered Cytotoxicity and Apoptosis by Mannan/β-Glucans from Yeast Cell Wall: Involvement of Autophagy and PI3K-AKT-MTOR Signaling Pathway. Int. J. Biol. Macromol..

[121] Song Y., Shen H., Liu T., Pan B., De Alwis S., Zhang W., Luo X., Li Z., Wang N., Ma W. (2021). Effects of Three Different Mannans on Obesity and Gut Microbiota in High-Fat Diet-Fed C57BL/6J Mice. Food Funct..

[122] Wang Y., Shen C., Huo K., Cai D., Zhao G. (2021). Antioxidant Activity of Yeast Mannans and Their Growth-Promoting Effect on Lactobacillus Strains. Food Funct..

[123] Li R., Zhu C., Bian X., Jia X., Tang N., Cheng Y. (2020). An Antioxidative Galactomannan Extracted from Chinese: Sesbania Cannabina Enhances Immune Activation of Macrophage Cells. Food Funct..

[124] Tao Y., Wang T., Huang C., Lai C., Ling Z., Yong Q. (2021). Effects of Seleno-Sesbania Canabina Galactomannan on Anti-Oxidative and Immune Function of Macrophage. Carbohydr. Polym..

[125] Castro B., Palomares T., Azcoitia I., Bastida F., del Olmo M., Soldevilla J.J., Alonso-Varona A. (2015). Development and Preclinical Evaluation of a New Galactomannan-Based Dressing with Antioxidant Properties for Wound Healing. Histol. Histopathol..

[126] Lima I.C., Castro R.R., Adjafre B.L., Sousa S.H.A.F., de Paula D.S., Alves A.P.N.N., Silva P.G.B., Assreuy A.M.S., Mota M.R.L. (2022). Galactomannan of Delonix Regia Seeds Modulates Cytokine Expression and Oxidative Stress Eliciting Anti-Inflammatory and Healing Effects in Mice Cutaneous Wound. Int. J. Biol. Macromol..

[127] Zhang D., Zhou X., Liu L., Guo M., Huang T., Zhou W., Geng F., Cui S.W., Nie S. (2021). Glucomannan From Aloe Vera Gel Promotes Intestinal Stem Cell-Mediated Epithelial Regeneration via the Wnt/β-Catenin Pathway. J. Agric. Food Chem..

[128] Lemieszek M.K., Nunes F.M., Rzeski W. (2019). Branched Mannans from the Mushroom: Cantharellus Cibarius Enhance the Anticancer Activity of Natural Killer Cells against Human Cancers of Lung and Colon. Food Funct..

[129] Lemieszek M.K., Nunes F.M., Marques G., Rzeski W. (2019). Cantharellus Cibarius Branched Mannans Inhibits Colon Cancer Cells Growth by Interfering with Signals Transduction in NF-ĸB Pathway. Int. J. Biol. Macromol..

[130] Tong X., Lao C., Li D., Du J., Chen J., Xu W., Li L., Ye H., Guo X., Li J. (2022). An Acetylated Mannan Isolated from Aloe Vera Induce Colorectal Cancer Cells Apoptosis via Mitochondrial Pathway. Carbohydr. Polym..

[131] Zhang K., Zhang D., Wang J., Wang Y., Hu J., Zhou Y., Zhou X., Nie S., Xie M. (2022). Aloe gel glucomannan induced colon cancer cell death via mitochondrial damage-driven PINK1/Parkin mitophagy pathway. Carbohydr. Polym..

[132] Chen J., Zhang X.D., Jiang Z. (2013). The application of fungal β-glucans for the treatment of colon cancer. Anticancer Agents Med. Chem..

[133] Jayachandran M., Chen J., Chung S.S.M., Xu B. (2018). A Critical Review on the Impacts of β-Glucans on Gut Microbiota and Human Health. J. Nutr. Biochem..

[134] Kopiasz Ł., Dziendzikowska K., Gajewska M., Wilczak J., Harasym J., Żyła E., Kamola D., Oczkowski M., Królikowski T., Gromadzka-Ostrowska J. (2020). Time-Dependent Indirect Antioxidative Effects of Oat Beta-Glucans on Peripheral Blood Parameters in the Animal Model of Colon Inflammation. Antioxidants.

[135] Taylor H.B., Gudi R., Brown R., Vasu C. (2020). Dynamics of Structural and Functional Changes in Gut Microbiota during Treatment with a Microalgal β-Glucan, Paramylon and the Impact on Gut Inflammation. Nutrients.

[136] Bai J., Zhao J., Al-Ansi W., Wang J., Xue L., Liu J., Wang Y., Fan M., Qian H., Li Y. (2021). Oat β-Glucan Alleviates DSS-Induced Colitis: Via Regulating Gut Microbiota Metabolism in Mice. Food Funct..

[137] Kopiasz Ł., Dziendzikowska K., Gromadzka-ostrowska J. (2022). Colon Expression of Chemokines and Their Receptors Depending on the Stage of Colitis and Oat Beta-Glucan Dietary Intervention—Crohn’s Disease Model Study. Int. J. Mol. Sci..

[138] Safwat El-Deeb O., El-Esawy R.O., Al-Shenawy H.A., Ghanem H.B. (2022). Modulating Gut Dysbiosis and Mitochondrial Dysfunction in Oxazolone-Induced Ulcerative Colitis: The Restorative Effects of β-Glucan and/or Celastrol. Redox Rep..

[139] Fahlquist-Hagert C., Sareila O., Rosendahl S., Holmdahl R. (2022). Variants of Beta-Glucan Polysaccharides Downregulate Autoimmune Inflammation. Commun. Biol..

[140] Silva N.A., Pereira B.G., Santos J.A., Guarnier F.A., Barbosa-Dekker A.M., Dekker R.F.H., Kassuya C.A.L., Bernardes S.S. (2022). Oral Administration of Botryosphaeran [(1 → 3)(1 → 6)-β-d-Glucan] Reduces Inflammation through Modulation of Leukocytes and Has Limited Effect on Inflammatory Nociception. Cell Biochem. Funct..

[141] Bahú J.O., de Andrade L.R.M., de Melo Barbosa R., Crivellin S., da Silva A.P., Souza S.D.A., Cárdenas Concha V.O., Severino P., Souto E.B. (2022). Plant Polysaccharides in Engineered Pharmaceutical Gels. Bioengineering.

[142] Wang X., He J., Pang S., Yao S., Zhu C., Zhao J., Liu Y., Liang C., Qin C. (2022). High-Efficiency and High-Quality Extraction of Hemicellulose of Bamboo by Freeze-Thaw Assisted Two-Step Alkali Treatment. Int. J. Mol. Sci..

[143] Sarker T.R., Pattnaik F., Nanda S., Dalai A.K., Meda V., Naik S. (2021). Hydrothermal pretreatment technologies for lignocellulosic biomass: A review of steam explosion and subcritical water hydrolysis. Chemosphere.

[144] Carvalheiro F., Duarte L.C., Gírio F.M. (2008). Hemicellulose biorefineries: A review on biomass pretreatments. J. Sci. Ind. Res..

[145] Yao S.Q., Nie S.X., Yuan Y., Wang S.F., Qin C.R. (2015). Efficient extraction of bagasse hemicelluloses and characterization of solid remainder. Bioresour. Technol..

[146] Wang Y., Cao X., Zhang R., Xiao L., Yuan T., Shi Q., Sun R. (2018). Evaluation of xylooligosaccharide production from residual hemicelluloses of dissolving pulp by acid and enzymatic hydrolysis. RSC Adv..

[147] Yuan Q., Liu S., Ma M.-G., Ji X.-X., Choi S.-E., Si C. (2021). The Kinetics Studies on Hydrolysis of Hemicellulose. Front. Chem..

[148] Wang X.H., Zhang C.H., Lin Q.X., Cheng B.G., Kong F.G., Li H.L., Ren J.L. (2018). Solid acid-induced hydrothermal treatment of bagasse for production of furfural and levulinic acid by a two-step process. Ind. Crop. Prod..

[149] Rehman A., Jafari S.M., Tong Q., Riaz T., Assadpour E., Aadil R.M., Niazi S., Khan I.M., Shehzad Q., Ali A. (2020). Drug nanodelivery systems based on natural polysaccharides against different diseases. Adv. Colloid Interface Sci..

[150] Wijaya C.J., Ismadji S., Gunawan S. (2021). A Review of Lignocellulosic-Derived Nanoparticles for Drug Delivery Applications: Lignin Nanoparticles, Xylan Nanoparticles, and Cellulose Nanocrystals. Molecules.

[151] Gupta A., Gupta G.S. (2022). Applications of Mannose-Binding Lectins and Mannan Glycoconjugates in Nanomedicine.

[152] Siemińska-Kuczer A., Szymańska-Chargot M., Zdunek A. (2022). Recent Advances in Interactions between Polyphenols and Plant Cell Wall Polysaccharides as Studied Using an Adsorption Technique. Food Chem..

[153] Loke Y.L., Chew M.T., Ngeow Y.F., Lim W.W.D., Peh S.C. (2020). Colon Carcinogenesis: The Interplay Between Diet and Gut Microbiota. Front. Cell. Infect. Microbiol..

[154] Kumar S., Negi Y.S. (2012). Corn Cob Xylan-Based Nanoparticles: Ester Prodrug of 5-Aminosalicylic Acid for Possible Targeted Delivery of Drug. J. Pharm. Sci. Res..

[155] Fu G.Q., Su L.Y., Yue P.P., Huang Y.H., Bian J., Li M.F., Peng F., Sun R.C. (2019). Syntheses of Xylan Stearate Nanoparticles with Loading Function from By-Products of Viscose Fiber Mills. Cellulose.

[156] Sauraj, Kumar S.U., Gopinath P., Negi Y.S. (2017). Synthesis and Bio-Evaluation of Xylan-5-Fluorouracil-1-Acetic Acid Conjugates as Prodrugs for Colon Cancer Treatment. Carbohydr. Polym..

[157] Urtiga S.C.d.C., Alves V.M.O., Melo C. (2020). de O.; Lima, M.N. de; Souza, E.; Cunha, A.P.; Ricardo, N.M.P.S.; Oliveira, E.E.; Egito, E.S.T. do. Xylan Microparticles for Controlled Release of Mesalamine: Production and Physicochemical Characterization. Carbohydr. Polym..

[158] Sauraj, Kumar V., Kumar B., Deeba F., Bano S., Kulshreshtha A., Gopinath P., Negi Y.S. (2019). Lipophilic 5-Fluorouracil Prodrug Encapsulated Xylan-Stearic Acid Conjugates Nanoparticles for Colon Cancer Therapy. Int. J. Biol. Macromol..

[159] Kowalska G., Rosicka-Kaczmarek J., Miśkiewicz K., Wiktorska M., Gumul D., Orczykowska M., Dędek K. (2021). Influence of Rye Bran Heteropolysaccharides on the Physicochemical and Antioxidant Properties of Honeydew Honey Microcapsules. Food Bioprod. Process..

[160] Kowalska G., Rosicka-Kaczmarek J., Miśkiewicz K., Zakłos-Szyda M., Rohn S., Kanzler C., Wiktorska M., Niewiarowska J. (2022). Arabinoxylan-Based Microcapsules Being Loaded with Bee Products as Bioactive Food Components Are Able to Modulate the Cell Migration and Inflammatory Response—In Vitro Study. Nutrients.

[161] Zhou X., Li W., Wang S., Zhang P., Wang Q., Xiao J., Zhang C., Zheng X., Xu X., Xue S. (2019). YAP Aggravates Inflammatory Bowel Disease by Regulating M1/M2 Macrophage Polarization and Gut Microbial Homeostasis. Cell Rep..

[162] Sauraj, Kumar S.U., Kumar V., Priyadarshi R., Gopinath P., Negi Y.S. (2018). PH-Responsive Prodrug Nanoparticles Based on Xylan-Curcumin Conjugate for the Efficient Delivery of Curcumin in Cancer Therapy. Carbohydr. Polym..

[163] Sauraj, Kumar V., Kumar B., Priyadarshi R., Deeba F., Kulshreshtha A., Kumar A., Agrawal G., Gopinath P., Negi Y.S. (2020). Redox Responsive Xylan-SS-Curcumin Prodrug Nanoparticles for Dual Drug Delivery in Cancer Therapy. Mater. Sci. Eng. C.

[164] Zhao B., Li H., Su Y., Tian K., Zou Z., Wang W. (2022). Synthesis and Anticancer Activity of Bagasse Xylan/Resveratrol Graft-Esterified Composite Nanoderivative. Materials.

[165] Gami P., Kundu D., Seera S.D.K., Banerjee T. (2020). Chemically Crosslinked Xylan–β-Cyclodextrin Hydrogel for the in Vitro Delivery of Curcumin and 5-Fluorouracil. Int. J. Biol. Macromol..

[166] Chimphango A.F.A., Matavire T.O. (2019). Performance and Structural Comparison of Hydrogels Made from Wheat Bran Arabinoxylan Using Enzymatic and Coacervation Methods as Micro-and Nano- Encapsulation and Delivery Devices. Biomed. Microdevices.

[167] Mendez-Encinas M.A., Carvajal-Millan E., Rascón-Chu A., Astiazarán-Garcia H., Valencia-Rivera D.E., Brown-Bojorquez F., Alday E., Velazquez C. (2019). Arabinoxylan-Based Particles: In Vitro Antioxidant Capacity and Cytotoxicity on a Human Colon Cell Line. Medicina.

[168] Bouramtane S., Bretin L., Pinon A., Leger D., Liagre B., Richard L., Brégier F., Sol V., Chaleix V. (2019). Porphyrin-Xylan-Coated Silica Nanoparticles for Anticancer Photodynamic Therapy. Carbohydr. Polym..

[169] Bretin L., Pinon A., Bouramtane S., Ouk C., Richard L., Perrin M., Chaunavel A., Carrion C. (2019). Photodynamic Therapy Activity of New Human Colorectal Cancer. Cancers.

[170] Su Y., Zhang S., Li H., Zhao B., Tian K., Zou Z. (2022). Dimethylaminoethyl Methacrylate/Diethylene Glycol Dimethacrylate Grafted onto Folate-Esterified Bagasse Xylan/Andrographolide Composite Nanoderivative: Synthesis, Molecular Docking and Biological Activity. Molecules.

[171] Tian K., Li H., Zhao B., Su Y., Zou Z., Wang W. (2022). Synthesis, Characterization and Bioactivity Evaluation of a Novel Nano Bagasse Xylan/Andrographolide Grafted and Esterified Derivative. Polymers.

[172] da Costa Urtiga S.C., de Lucena Gabi C.A.A., de Araújo Eleamen G.R., Santos Souza B., de Luna Freire Pessôa H., Marcelino H.R., de Moura Mendonça E.A., do Egito E.S.T., Oliveira E.E. (2017). Preparation and Characterization of Safe Microparticles Based on Xylan. Drug Dev. Ind. Pharm..

[173] Santos M.B., Garcia-Rojas E.E. (2021). Recent Advances in the Encapsulation of Bioactive Ingredients Using Galactomannans-Based as Delivery Systems. Food Hydrocoll..

[174] Meng F.B., Zhang Q., Li Y.C., Li J.J., Liu D.Y., Peng L.X. (2020). Konjac Glucomannan Octenyl Succinate as a Novel Encapsulation Wall Material to Improve Curcumin Stability and Bioavailability. Carbohydr. Polym..

[175] Wang L.H., Xiao J.X., Li X.D., Huang G.Q. (2021). Carboxymethyl Konjac Glucomannan Coating on Multilayered Emulsions for Improved Bioavailability and Targeted Delivery of Curcumin. Food Funct..

[176] Wu C., Sun J., Jiang H., Li Y., Pang J. (2021). Construction of Carboxymethyl Konjac Glucomannan/Chitosan Complex Nanogels as Potential Delivery Vehicles for Curcumin. Food Chem..

[177] Nguyen Q.D., Dang T.T., Nguyen T.V.L., Nguyen T.T.D., Nguyen N.N. (2022). Microencapsulation of Roselle (*Hibiscus sabdariffa* L.) Anthocyanins: Effects of Different Carriers on Selected Physicochemical Properties and Antioxidant Activities of Spray-Dried and Freeze-Dried Powder. Int. J. Food Prop..

[178] de Almeida Paula D., Mota Ramos A., Basílio de Oliveira E., Maurício Furtado Martins E., Augusto Ribeiro de Barros F., Cristina Teixeira Ribeiro Vidigal M., de Almeida Costa N., Tatagiba da Rocha C. (2018). Increased Thermal Stability of Anthocyanins at PH 4.0 by Guar Gum in Aqueous Dispersions and in Double Emulsions W/O/W. Int. J. Biol. Macromol..

[179] Kuck L.S., Noreña C.P.Z. (2016). Microencapsulation of Grape (Vitis Labrusca Var. Bordo) Skin Phenolic Extract Using Gum Arabic, Polydextrose, and Partially Hydrolyzed Guar Gum as Encapsulating Agents. Food Chem..

[180] Pieczykolan E., Kurek M.A. (2019). Use of Guar Gum, Gum Arabic, Pectin, Beta-Glucan and Inulin for Microencapsulation of Anthocyanins from Chokeberry. Int. J. Biol. Macromol..

[181] Samborska K., Boostani S., Geranpour M., Hosseini H., Dima C., Khoshnoudi-Nia S., Rostamabadi H., Falsafi S.R., Shaddel R., Akbari-Alavijeh S. (2021). Green Biopolymers from By-Products as Wall Materials for Spray Drying Microencapsulation of Phytochemicals. Trends Food Sci. Technol..

[182] Liu J., Chen F., Tian W., Ma Y., Li J., Zhao G. (2014). Optimization and Characterization of Curcumin Loaded in Octenylsuccinate Oat β-Glucan Micelles with an Emphasis on Degree of Substitution and Molecular Weight. J. Agric. Food Chem..

[183] Liu J., Lei L., Ye F., Zhou Y., Younis H.G.R., Zhao G. (2018). Aggregates of Octenylsuccinate Oat β-Glucan as Novel Capsules to Stabilize Curcumin over Food Processing, Storage and Digestive Fluids and to Enhance Its Bioavailability. Food Funct..

[184] Plavcová Z., Šalamúnová P., Saloň I., Štěpánek F., Hanuš J., Hošek J. (2019). Curcumin Encapsulation in Yeast Glucan Particles Promotes Its Anti-Inflammatory Potential in Vitro. Int. J. Pharm..

[185] Rotrekl D., Šalamúnová P., Paráková L., Baďo O., Saloň I., Štěpánek F., Hanuš J., Hošek J. (2021). Composites of Yeast Glucan Particles and Curcumin Lead to Improvement of Dextran Sulfate Sodium-Induced Acute Bowel Inflammation in Rats. Carbohydr. Polym..

[186] Singh A., Lavkush, Kureel A.K., Dutta P.K., Kumar S., Rai A.K. (2018). Curcumin Loaded Chitin-Glucan Quercetin Conjugate: Synthesis, Characterization, Antioxidant, in Vitro Release Study, and Anticancer Activity. Int. J. Biol. Macromol..

[187] Šalamúnová P., Cupalová L., Majerská M., Treml J., Ruphuy G., Šmejkal K., Štěpánek F., Hanuš J., Hošek J. (2021). Incorporating Natural Anti-Inflammatory Compounds into Yeast Glucan Particles Increases Their Bioactivity in Vitro. Int. J. Biol. Macromol..

[188] Feng X., Xie Q., Xu H., Zhang T., Li X., Tian Y., Lan H., Kong L., Zhang Z. (2022). Yeast Microcapsule Mediated Natural Products Delivery for Treating Ulcerative Colitis through Anti-Inflammatory and Regulation of Macrophage Polarization. ACS Appl. Mater. Interfaces.

[189] Ahmad M., Ashraf B., Gani A., Gani A. (2018). Microencapsulation of Saffron Anthocyanins Using β Glucan and β Cyclodextrin: Microcapsule Characterization, Release Behaviour & Antioxidant Potential during in-Vitro Digestion. Int. J. Biol. Macromol..

